# Sialidases Affect the Host Cell Adherence and Epsilon Toxin-Induced Cytotoxicity of *Clostridium perfringens* Type D Strain CN3718

**DOI:** 10.1371/journal.ppat.1002429

**Published:** 2011-12-08

**Authors:** Jihong Li, Sameera Sayeed, Susan Robertson, Jianming Chen, Bruce A. McClane

**Affiliations:** 1 Department of Microbiology and Molecular Genetics, University of Pittsburgh School of Medicine, Pittsburgh, Pennsylvania, United States of America; 2 Department of Environmental and Occupational Health, University of Pittsburgh, Pennsylvania, United States of America; The University of Texas-Houston Medical School, United States of America

## Abstract

*Clostridium perfringens* type B or D isolates, which cause enterotoxemias or enteritis in livestock, produce epsilon toxin (ETX). ETX is exceptionally potent, earning it a listing as a CDC class B select toxin. Most *C. perfringens* strains also express up to three different sialidases, although the possible contributions of those enzymes to type B or D pathogenesis remain unclear. Type D isolate CN3718 was found to carry two genes (*nanI* and *nanJ*) encoding secreted sialidases and one gene (*nanH*) encoding a cytoplasmic sialidase. Construction in CN3718 of single *nanI, nanJ* and *nanH* null mutants, as well as a *nanI*/*nanJ* double null mutant and a triple sialidase null mutant, identified NanI as the major secreted sialidase of this strain. Pretreating MDCK cells with NanI sialidase, or with culture supernatants of BMC206 (an isogenic CN3718 *etx* null mutant that still produces sialidases) enhanced the subsequent binding and cytotoxic effects of purified ETX. Complementation of BMC207 (an *etx/nanH/nanI/nanJ* null mutant) showed this effect is mainly attributable to NanI production. Contact between BMC206 and certain mammalian cells (e.g., enterocyte-like Caco-2 cells) resulted in more rapid sialidase production and this effect involved increased transcription of BMC206 *nanI* gene. BMC206 was shown to adhere to some (e.g. Caco-2 cells), but not all mammalian cells, and this effect was dependent upon sialidase, particularly NanI, expression. Finally, the sialidase activity of NanI (but not NanJ or NanH) could be enhanced by trypsin. Collectively these *in vitro* findings suggest that, during type D disease originating in the intestines, trypsin may activate NanI, which (in turn) could contribute to intestinal colonization by *C. perfringens* type D isolates and also increase ETX action.

## Author Summary

*Clostridium perfringens* type D strains cause enteritis and enterotoxemias in livestock after colonizing the intestines and then producing toxins, notably epsilon toxin (ETX). Initially produced and secreted in an inactive form, ETX can be rapidly proteolytically-activated by trypsin and other intestinal proteases. While most *C. perfringens* strains produce three sialidases, no pathogenic role has yet been identified for these enzymes that remove terminal sialic acid residues from glycoproteins and glycolipids. Our current study found that trypsin increases the activity of the NanI sialidase made by type D strain CN3718. This effect enhanced the ability of NanI to modify the surface of MDCK cells, leading to increased ETX binding and cytotoxicity. We also found that modification of the host cell surface by NanI sialidase allows efficient attachment of CN3718 cells to Caco-2 cells. These results identify interactions between intestinal proteases, ETX, sialidases, and ETX-producing bacteria, whereby trypsin activates not only ETX but also NanI sialidase. If similar effects occur in the intestines, the activated NanI sialidase may modify the host cell surface to facilitate bacterial attachment and thereby worsen disease by facilitating intestinal colonization by type D strains to prolong toxin delivery and, in some species, increase ETX binding.

## Introduction

*Clostridium perfringens*, a Gram-positive, spore-forming anaerobe, is an important pathogen of both humans (causing, for example, gas gangrene and type A human food poisoning) and livestock (causing severe enterotoxemias and enteritis) [1]. The virulence of this bacterium is largely attributable to its ability to express a plethora of potent toxins. However, while *C. perfringens* can produce >15 different toxins, individual strains express only portions of this toxin arsenal [1]-[3]. Therefore, based upon production of four typing toxins (α, β, ε, and ι), isolates of this organism are commonly classified into five toxinotypes (type A through E) [4].

By definition, *C. perfringens* type D isolates must produce alpha and epsilon toxins, while type B isolates must express alpha, beta and epsilon toxin [4]. Beyond those typing toxins, type D and type B isolates commonly produce additional toxins, e.g., perfringolysin O, enterotoxin, TpeL or beta2 toxin [5]-[7]. Type B and D isolates cause enterotoxemias in livestock that initiate with toxin production in the intestines, followed by absorption of those toxins into the circulation to affect other internal organs, such as the brain and kidneys. Type D isolates can also cause acute or chronic enteritis in goats [1], [8], [9].

Epsilon toxin (ETX) is considered important for the virulence of both type B and type D isolates [5], [6]. This CDC class B select toxin, which ranks as the third most-potent clostridial toxin after the botulinum toxins and tetanus toxin, belongs to the aerolysin family of pore-forming toxins [9], [10]. ETX is synthesized and secreted as an inactive prototoxin of 311 amino acids (32.7 kDa). In the animal intestines, the prototoxin can be proteolytically activated to the fully-active toxin (274 amino acids) by trypsin and chymotrypsin [11].

To date, only a few ETX-sensitive cultured cell lines have been identified, including MDCK cells, mpkCCDc14 cells, and human leiomyoblastoma (G402) cells [11]. The mechanism of ETX action on MDCK cells is still under active study but first involves the binding of this toxin to unidentified protein receptors on the MDCK cell membrane surface. The bound toxin then uses lipid rafts to form a heptameric prepore complex on the membrane surface [12], [13]. When this prepore complex inserts into the cell membrane, an active pore is created that causes, or strongly contributes to, MDCK cell death [9].

Genome sequencing has revealed that *C. perfringens* strains typically possess three sialidase-encoding genes, named *nanH*, *nanI* and *nanJ*, which are located on a conserved region of the chromosome [14]-[16]. The *nanH* gene product, which is not secreted, is the ∼43 kDa NanH sialidase. The *nanI* gene product is the secreted ∼77 kDa NanI sialidase, while the *nanJ* gene product is the secreted ∼129 kDa NanJ sialidase. The *nanH* ORF shares only 19% nucleotide sequence identity with *nanI* and *nanJ*, but the ORFs encoding the two larger exoenzymes are more closely related, sharing 57% nucleotide sequence identity [17].

Sialidase (neuraminidases) production by some pathogenic bacteria has been implicated in their virulence. For example, *Vibrio cholerae* sialidase enhances the activity of cholera toxin by modifying surface gangliosides to create additional toxin receptors and thereby increase toxin binding levels [18]. Sialidases can also apparently contribute to virulence in other ways besides enhancing toxin binding. For example, neuramindases are thought to assist *Streptococcus pneumoniae* pathogenesis by providing nutrients for growth, assisting in biofilm formation, and enhancing colonization by exposing adhesion sites for this bacterium in the airways [19].

Possible sialidase contributions to *C. perfringens* virulence have received only limited attention. A recent study [20] used mutants to evaluate the potential pathogencity contributions of NanI and NanJ sialidase when *C. perfringens* type A strain 13 causes clostridial myonecrosis (gas gangrene). That study [20] found sialidases can enhance alpha-toxin-mediated cytotoxic effects *in vitro,* but also reported that sialidase production is not necessary for strain 13 to cause gas gangrene in a mouse model. Possible virulence contributions of sialidases when other *C. perfringens* strains, such as type D strains, cause disease originating in the intestines has received even less study. It was reported that sialidases can enhance ETX cytotoxicity towards MDCK cells [21], but an apparently contradictory conclusion was reached by another study determining that treatment of synaptosomal membrane fractions with sialidases lowered ETX binding levels [22].

The current study has used both biochemical and isogenic mutant approaches to better evaluate the possible sialidase enhancement of *in vitro* ETX action. In addition, a possible role for sialidases in facilitating *C. perfringens* cell adhesion to host cells was examined for the first time. Results of these *in vitro* studies suggest that sialidases could contribute to type D pathogenesis via at least two distinct mechanisms.

## Results

### Pretreatment of MDCK cells with purified *C. perfringens* NanI sialidase enhances ETX cytotoxicity, binding and complex formation

**Figure 1:**
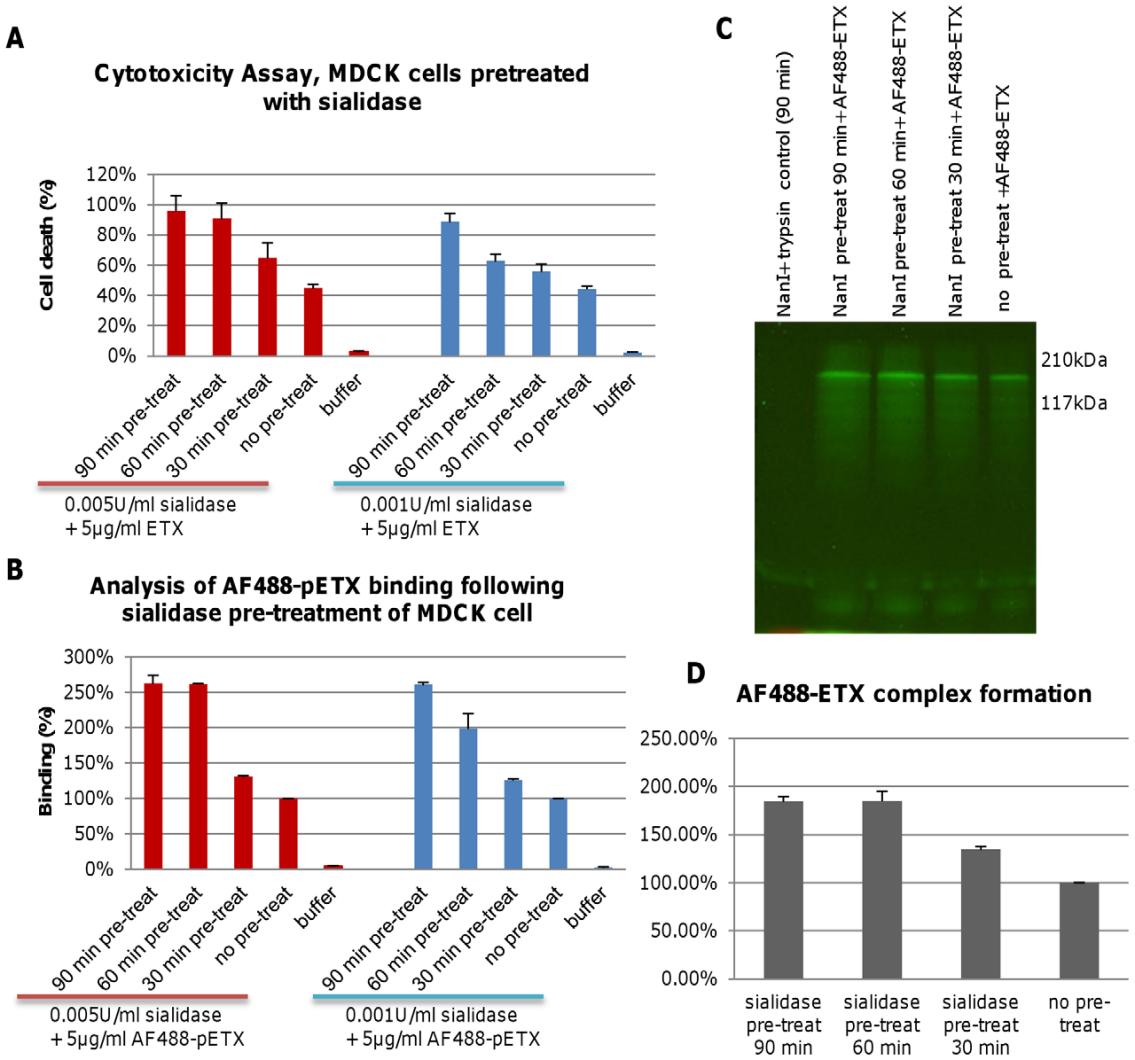
Pre-treatment of MDCK cells with purified NanI enhances ETX binding and cytotoxicity. Panel A, cytotoxicity assay of MDCK cells pretreated for the specified times with either 0.001 U/ml or 0.005 U/ml of NanI sialidase and then challenged with 5 µg/ml of purified ETX. Panel B, AF488-pETX binding following the specified sialidase pre-treatment of MDCK cells. AF488-pETX (5 µg/ml) binding to MDCK cells in the absence of any pre-treatment with sialidase was considered 100% binding. Panel C, pre-treatment with purified NanI sialidase enhances AF488-ETX complex formation in, MDCK cells. Lane 1, sialidase and trypsin controls; Lane 2-4, ETX complex formation after sialidase pre-treatment of MDCK cells for 30, 60 or 90 min, followed by treatment with 5 µg/ml of AF488-ETX. Lane 5, ETX complex formation by 5 µg/ml AF488-ETX-treated MDCK cells (no sialidase pretreatment); Molecular weight markers shown on the right. Panel D, quantitative comparison of the effects of NanI pretreatment on AF488-ETX complex formation. Gel regions from panel C experiments containing the ETX complex were scanned for fluorescence using a Typhoon scanner. The results shown in Figure 1 show the average of two (panel D) or three (panel A and B) repetitions; the error bars indicate the standard deviation (S.D.).

To clarify whether *C. perfringens* sialidases can enhance ETX cytotoxicity in sensitive mammalian cells, MDCK cells were pretreated with purified *C. perfringens* NanI sialidase and, after washing, those cells were challenged with purified ETX. The results showed that sialidase pretreatment substantially increased ETX-induced cytotoxicity (Figure 1A). This enhancement was stronger using a 0.005 U/ml vs. a 0.001 U/ml dose of sialidase for the pretreatment. However, while enhancement of ETX cytotoxicity increased up to a 90 min sialidase pretreatment using the 0.001 U/ml NanI dose, the higher 0.005 U/ml NanI dose caused no further increase in cytotoxicity beyond a 60 min pretreatment (Figure 1A). Sialidase treatment alone, or treatment with prototoxin, did not increase cytotoxicity above background levels (data not shown).

Experiments were then performed to explore the mechanistic basis behind the sialidase-induced enhancement of ETX cytotoxicity for MDCK cells that was shown in Figure 1A. First, MDCK cells were pretreated with purified *C. perfringens* NanI sialidase and, after washing, those cells were incubated with Alexa Fluor 488-labeled epsilon prototoxin (AF488-pETX), which was used since it binds similarly as active ETX to MDCK cells, yet causes no cytotoxicity [23]. Results from this experiment (Figure 1B) showed that pretreating MDCK cells with either 0.001 U/ml or 0.005 U/ml of purified NanI sialidase substantially increased subsequent AF488-pETX binding levels. This enhancement of toxin binding increased with longer sialidase pretreatment time, although no further increase in AF488-pETX binding was noted after a 60 min sialidase pretreatment beyond the 0.005 U/ml sialidase dose (Figure 1B).

An experiment then assessed the ability of sialidase pretreatment to increase formation of the ETX oligomeric complex that is considered responsible for pore formation-induced ETX cytotoxicity [9]. SDS-PAGE analyses of MDCK cell lysates treated with 5 µg/ml of Alexa Fluor 488-labeled ETX (AF488-ETX) detected an increased formation of the ETX oligomeric complex after NanI pretreatment (Figure 1C and 1D). This effect increased up to a 60 min pretreatment with 0.005 U/ml of NanI. Lastly, similar NanI pretreatment also increased MDCK cell binding and complex formation using a 10 µg/ml dose of AF488-pETX or AF488-ETX, respectively (data not shown).

### Construction and genotypic characterization of isogenic CN3718 sialidase mutants

**Figure 2:**
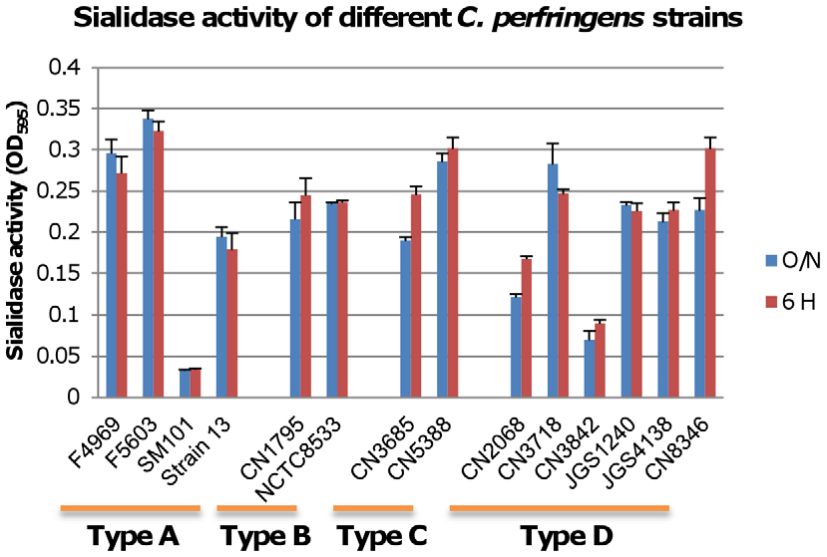
Sialidase activity in different type *C. perfringens* strains. Sialidase activity was measured in supernatants from overnight and 6 h TH cultures of the specified *C. perfringens* strains, including type A strains F4969, F5603, SM101and Strain13; type B strains CN1795 and NCTC8533; type C strains CN3685 and CN5385 and type D strains CN2068, CN3718, CN3842, JGS1240, JGS4138 and CN8346. Results shown are the average of three repetitions; the error bars indicate the standard deviation (S.D.). The origin and typing of these strains has been described previously [14], [16], [33]-[36].

The Figure 1 results indicated that pretreating MDCK cells with purified *C. perfringens* NanI sialidase can enhance ETX cytotoxicity by increasing toxin binding and thus, complex formation. Therefore, we next sought to assess whether sialidases also contribute to ETX-induced cytotoxicity when these glycoside hydrolyases are expressed at natural levels and in the natural presence of other *C. perfringens* exoproducts, including other toxins. To initiate this work, we first surveyed sialidase activity in 6 h and overnight supernatants from cultures of various *C. perfringens* isolates (Figure 2). This survey detected considerable strain-to-strain variations in supernatant sialidase activity and also identified type D strain CN3718 as a moderately high sialidase producer. Since CN3718 is also transformable and produces ETX (see below), it was chosen for construction of sialidase mutants.

PCR analyses first determined that CN3718 carries all three identified *C. perfringens* sialidase genes (data not shown), including *nanI* and *nanJ*, which encode the secreted NanI and NanJ sialidases, and *nanH*, which encodes the NanH sialidase that lacks a signal peptide and thus localizes in the cytoplasm. Therefore, the current study used a *Clostridium*-modified Targetron insertional mutagenesis method [24] to construct isogenic single *nanH*, *nanI*, and *nanJ* null mutants, a *nanI/nanJ* double null mutant, and a *nanH*/*nanI*/*nanJ* triple null mutant in a CN3718 background.

The identity of the CN3718 *nanJ*, *nanI* and *nanH* single null mutants (named BMC201, BMC202 and BMC203, respectively) was first demonstrated by PCR using primers specific for internal *nanJ* ORF, *nanI* ORF or *nanH* ORF sequences. Using DNA from wild-type CN3718, these internal PCR primers specifically amplified the expected PCR products of 306 bp for *nanJ*, 467 bp for *nanI*, and 285 bp for *nanH* (data not shown). However, consistent with the insertion of an ∼900 bp intron into the target ORF, the same primers amplified PCR products of ∼1200 bp, ∼1400 bp, and ∼1200 bp for the *nanJ, nanI* and *nanH* null mutants, respectively (data not shown). Those PCR assays also confirmed the identity of a *nanJ/I* double null mutant (BMC204) and a *nanJ/I/H* triple null mutant (BMC205) constructed by Targetron technology (data not shown). The *nanJ/I* double null mutant PCR supported amplification of two larger bands, which matched the product sizes of a disrupted *nanJ* gene and *nanI* gene, along with a small band matching the ∼285 bp product size for the wild-type *nanH* gene (data not shown). Using BMC205 DNA, PCR amplified larger bands for all three sialidase ORFs (data not shown).

**Figure 3:**
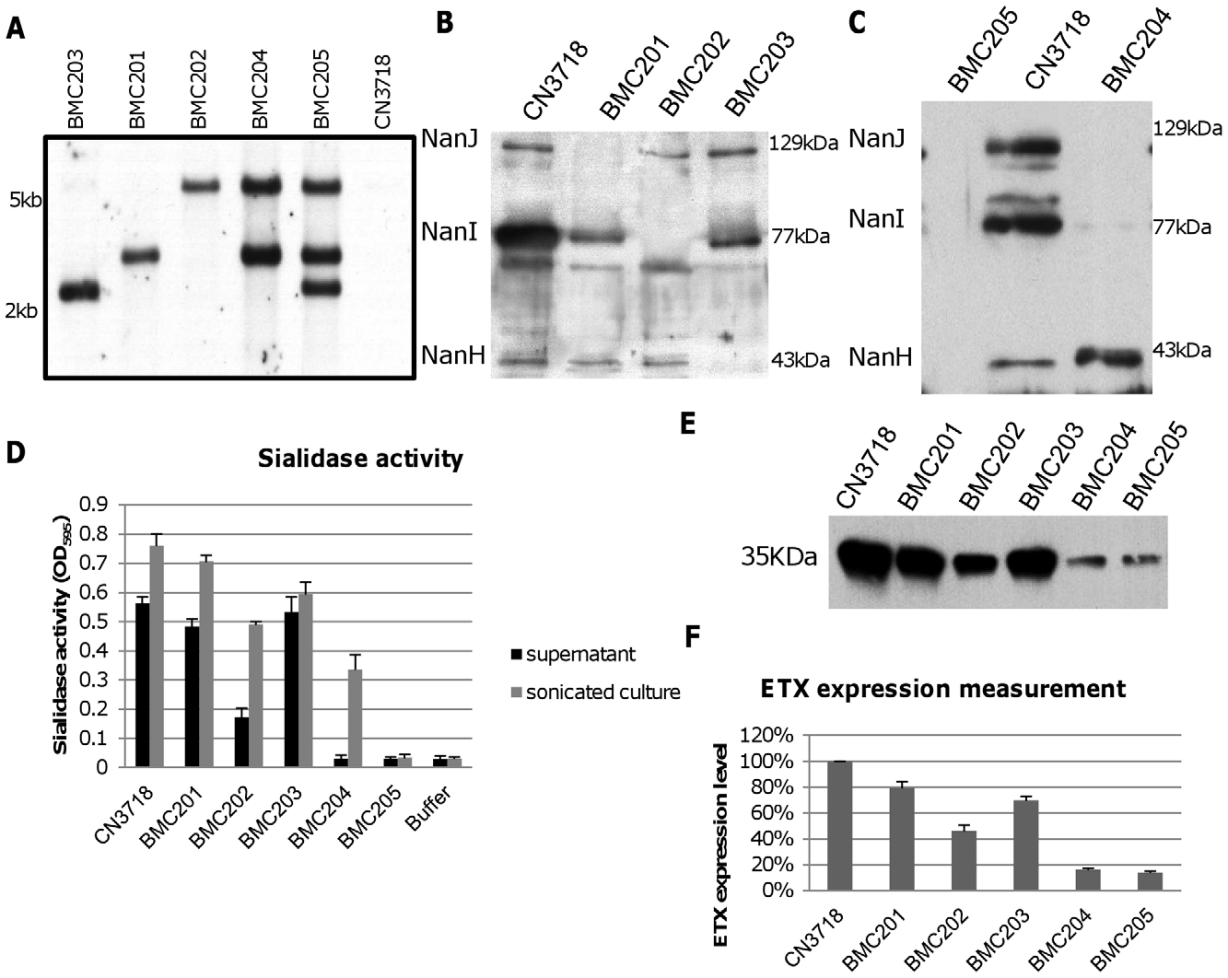
Intron-based mutagenesis to create isogenic CN3718 sialidase null mutants and characterization of those mutants. Panel A, Southern blot hybridization of an intron-specific probe with DNA from wild-type CN3718 or the sialidase null mutants (BMC201, BMC202, BMC203, BMC204 or BMC205). DNA from each strain was digested with BsrGI and electrophoresed on a 1% agarose gel prior to blotting and hybridization with an intron-specific probe. Size of DNA fragments, in kilobases (kb) is shown at left. Panel B, Western blot analyses for sialidase expression by wild-type CN3718 or the *nanJ*, *nanI* or *nanH* single null mutants named BMC201, BMC202, or BMC203, respectively. Size of proteins in kDa is shown at right. Panel C, Western blot analyses for sialidase expression by wild-type CN3718, the *nanJ*/*nanI* double null mutant (BMC204) or the *nanJ*/*nanI*/*nanH* triple null mutant (BMC205). Size of proteins in kDa is shown at right. Panel D, sialidase activity analyses for wild-type CN3718 or specified sialidase null mutant strains using 8 h TH culture supernatants or supernatants from 8 h TH sonicated cultures. Panel E, Western blot analyses for ETX expression by wild type CN3718 or sialidase single, double or triple null mutant strains (BMC201, BMC202, BMC203, BMC204 or BMC205). Size of prototoxin in kDa is shown at left. Panel F, Measurement of ETX expression by wild-type CN3718 or sialidase null mutant strains, based upon densitometric scanning of panel E gels. All the results show the average of three repetitions; the error bars indicate the standard deviation (S.D.).

After curing the intron delivery plasmids from the BMC201, BMC202, BMC203, BMC204 or BMC205 sialidase null mutants, DNA from the wild-type strain and each null mutant was subjected to Southern blot analysis (Figure 3A) using an intron-specific probe [25]. No probe hybridization to wild-type DNA was detected, as expected. In contrast, the presence of a single intron insertion was visible on this Southern blot using DNA from each single sialidase null mutant. In addition, two intron insertions were detected using DNA from the BMC204 *nanI/J* double null mutant and three intron insertions were noted using DNA from the BMC205 *nanI/J/H* triple null mutant.

### Phenotypic characterization of wild-type CN3718 and isogenic sialidase null mutants

To initiate a phenotypic characterization, vegetative growth of the isogenic mutants was compared in Todd Hewitt (TH) medium. After similar inoculation into TH medium, the vegetative growth rate of the isogenic mutants was nearly identical to the CN3718 parent (data not shown). Western blots then compared sialidase expression between CN3718 and the isogenic single, double and triple sialidase null mutants. These analyses demonstrated that sonicated 8 h TH cultures of the wild-type strain contain the 129 kDa NanJ, the 77 kDa NanI and the 43 kDa NanH (Figure 3B). In contrast, the BMC201 null mutant only produced NanI and NanH, the BMC202 null mutant only produced NanJ and NanH, and the BMC203 null mutant produced only NanI and NanJ (Figure 3B). Similar Western blotting of sonicated cultures (Figure 3C) showed that the BMC204 *nanI/J* double null mutant only produced the 43 kDa NanH, while the BMC205 *nanJ/I/H* triple null mutant did not produce any sialidase protein.

To evaluate the effects of mutating each sialidase gene on the total sialidase activity of CN3718, sialidase activity assays were carried out using either 8 h TH culture supernatants (to measure exosialidase activity) or sonicated 8 h TH culture supernatants (to measure total sialidase activity) from the wild-type parent and each null mutant strain. Collectively, the results (Figure 3D) indicated that the exosialidase activity measured in supernatants from the wild-type strain is mainly attributable to NanI. The Figure 3D results also confirmed that NanH predominantly accumulates in the cytoplasm since 8 h supernatants of the BMC204 *nanI/J* double mutant exhibited almost no sialidase activity, while the sonicated culture supernatant of this double mutant possessed significant sialidase activity. Lastly, this analysis demonstrated that CN3718 produces only the three recognized sialidases since the supernatant and sonicated culture of the BMC205 *nanI/J/H* triple null mutant lacked sialidase activity.

Since a major goal of the current study was to evaluate a possible relationship between sialidases and ETX action, ETX expression was measured for wild-type CN3718 and the isogenic sialidase null mutants. Surprisingly, each single sialidase mutant, but especially the BMC202 *nanI* null mutant, showed decreased ETX expression compared to the wild-type parent (Figure 3E and 3F). ETX expression levels by the BMC204 *nanI/J* double null mutant and the BMC205 *nanI/J/H* triple mutant decreased further compared against ETX production levels by wild-type CN3718 (Figure 3E and 3F).

### Construction and genotypic characterization of isogenic *etx* null mutants of CN3718 and BMC205

**Figure 4:**
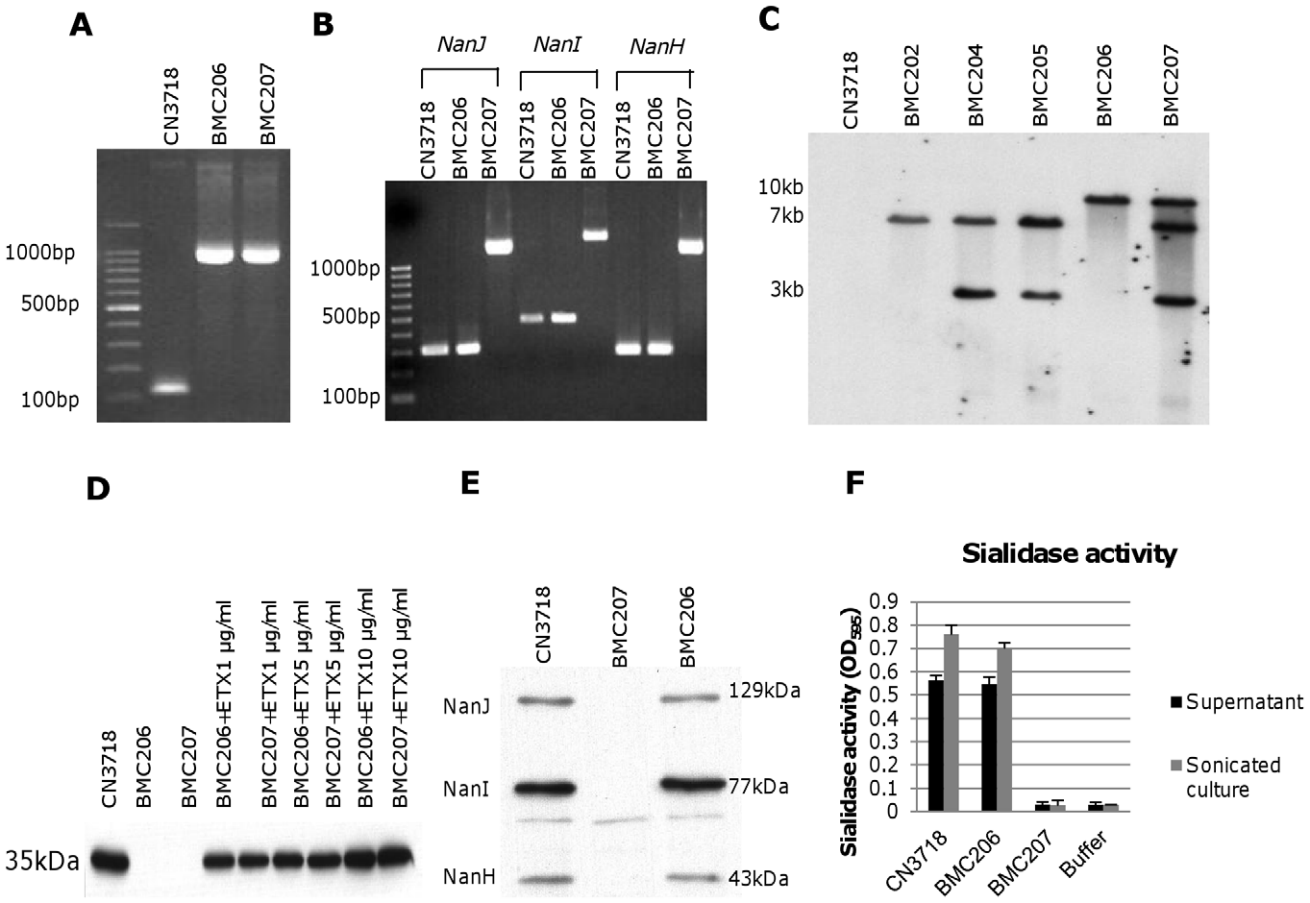
Intron-based mutagenesis to create *etx* null mutants of wild-type CN3718 and BMC205. Panel A, PCR confirmation of construction of isogenic *etx* null mutants of wild-type CN3718 (creating BMC206) and BMC205 (creating BMC207). Using DNA isolated from wild-type CN3718, a PCR assay amplified a 117 bp product using internal *etx* primers but an ∼1.0 kb product, consistent with insertion of a 0.9 kb intron, using template DNA isolated from BMC206 or BMC207 *etx* null mutants. Lane 1, 100 bp molecular size markers. Panel B, PCR assays for *nanI*, *nanJ* or *nanH* confirming that BMC206 carries the wild-type *nanI*, *nanJ* or *nanH* genes, but BMC207 has a 0.9 kb intron insertions in its *nanI*, *nanJ* and *nanH* null mutant genes. Lane 1, 100 bp molecular size markers. Panel C, Southern blot hybridization of an intron-specific probe with DNA from wild-type CN3718, BMC202, BMC204, BM205, BMC206 or BMC207. DNA from each strain was digested with EcoRI and electrophoresed on a 1% agarose gel prior to blotting and hybridization with an intron-specific probe. Size of DNA fragments, in kilobases (kb), is shown at left. Panel D, ETX Western blot analyses of supernatants from wild-type CN3718 or the BMC206 and BMC207 *etx* null mutants, without or with supplementation with 1 µg/ml, 5 µg/ml or 10 µg/ml of ETX. Size of prototoxin in kDa is shown at left. Panel E, Western blot analyses for sialidase expression by wild-type CN3718, BMC206 or BMC207. Size of proteins in kDa is shown at right. Panel F, sialidase activity analyses for wild-type CN3718 or the BMC206 and BMC207 *etx* null mutant strains using 8 h TH culture supernatants or supernatants from sonicated 8 h TH cultures.

The considerable ETX expression differences between wild-type strain CN3718 and the sialidase mutants shown in Figure 3E and 3F precluded using those strains for comparative ETX cytotoxicity experiments aimed at evaluating sialidase contributions to ETX action under natural sialidase expression levels and in the presence of natural levels of other secreted *C. perfringens* products. To overcome this complication, *etx* null mutations were engineered into the CN3718 wild-type strain and the BMC205 triple sialidase null mutant strain to create BMC206 and BMC207, respectively. The availability of these two *etx* mutants would allow later comparative cytotoxicity experiments using equivalent amounts of ETX added to supernatants of these strains. These *etx* mutants were constructed by a Targetron insertional mutagenesis approach. PCR confirmed the presence of an intron insertion into the *etx* gene (Figure 4A). Using DNA from wild-type CN3718, internal *etx* PCR primers specifically amplified a PCR product of 117 bp. However, consistent with a 900 bp intron insertion into the *etx* ORF, the same primers amplified a PCR product of ∼1000 bp using DNA from the putative *etx* mutants of both BMC206 and BMC207. PCR approaches also confirmed that BMC207 maintains intron insertions in its *nanI*, *nanJ* and *nanH* ORFs (Figure 4B).

After curing the intron delivery plasmids from these mutants, DNA was isolated from the two putative *etx* null mutants and subjected to Southern blotting using an intron-specific probe (Figure 4C). This analysis detected no probe hybridization with wild-type CN3718 DNA, as expected. As no suitable enzyme was identified that could separate the four intron copies present in BMC207, EcoRI was used to distinguish the presence of the *etx* intron from the other three sialidase introns, whose presence in the BMC205 mutant had already been demonstrated (Figure 3A). By this approach, the presence of the *etx* intron was clearly shown in BMC206 and BMC207 by Southern blotting (Figure 4C).

### Phenotypic characterization of isogenic BMC206 and BMC207 null mutants

To initiate phenotypic comparisons, the growth rates of the BMC206 and BMC207 *etx* null mutants were first determined to be similar to wild-type CN3718 in TH medium (data not shown). Western blotting then confirmed the lack of ETX expression by these *etx* null mutants (Figure 4D). For later experiments it was important to demonstrate that culture supernatants could be supplemented with purified ETX to achieve equivalent ETX levels. This was confirmed by adding 1, 5 or 10 µg/ml of ETX to supernatants of the two *etx* null mutants (Figure 4D).

Sialidase Western blotting and sialidase activity analyses (Figure 4E and 4F) were next performed using cultures or sonicated cultures (to release NanH) of wild-type CN3718, BMC206 and BMC207 to evaluate whether introducing an *etx* gene mutation had affected sialidase expression. Western blot results demonstrated comparable levels of NanJ, NanI and NanH production by wild-type CN3718 and BMC206 when grown for 8 h in TH. However, as expected, production of these three sialidases was absent from similar cultures of BMC207 (Figure 4E). Measurement of sialidase activity in sonicated cultures (Figure 4F) further confirmed those Western blotting results. The sialidase activity detected in TH sonicated lysates or TH culture supernatants of CN3718 vs. BMC206 was very similar. However, no sialidase activity was measured in similarly prepared BMC207 sonicated lysates or culture supernatants.

### Construction and characterization of *nanJ*, *nanI* and *nanH* complementing strains of BMC207

**Figure 5:**
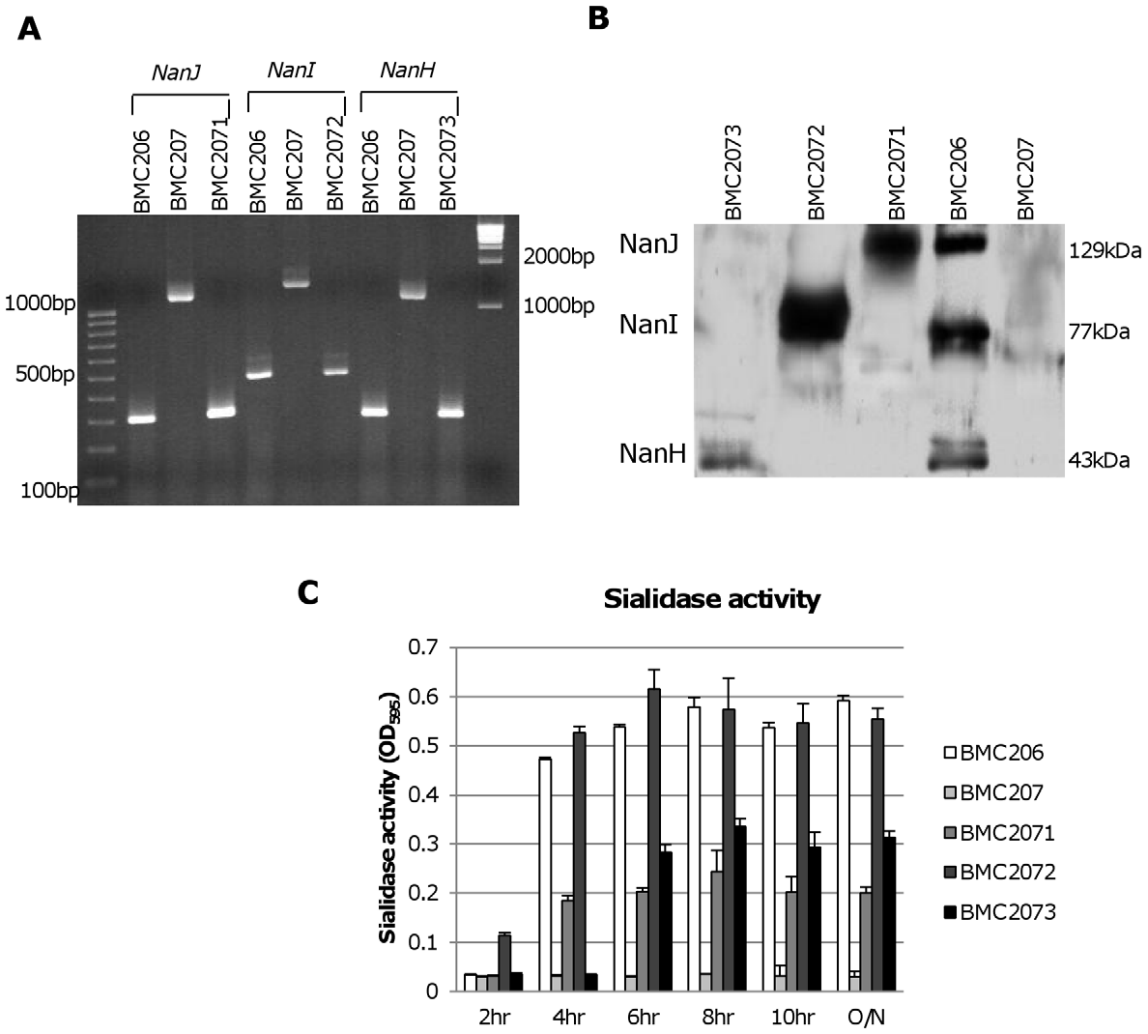
Characterization of sialidase complementing strains. Panel A, PCR confirmation of the construction of isogenic sialidase null mutant and complementing strains. Using DNA isolated from BMC206, PCR assays using internal sialidase gene primers amplified the expected wild-type *nanJ*, *nanI* and *nanH* products of 306 bp, 467 bp and 285 bp, respectively. Using template DNA isolated from BMC207, which has a 900 bp intron insertion in each sialidase gene, the same PCR assays amplified larger products of 1206 bp, 1367 and 1185 for *nanJ*, *nanI* and *nanH*, respectively. With the same assay, DNA from each complementing strain yielded the same wild-type sized PCR products as amplified from BMC206. Lines 1 and 11, 100 bp or 1 kb molecular weight markers, respectively. Panel B, Western blot analyses for sialidase expression by complementing strains BMC2071 (for *nanJ* complementation), BMC2072 (for *nanI* complementation) and BMC2073 (for *nanH* complementation) using 8 h TH sonicated cultures. Size of protein in kDa is shown at right. Panel C, sialidase activity detected in supernatants of BMC206, BMC207, BMC2071, BMC2072 or BMC2073 after growth in TH medium for 2 h, 4 h, 6 h, 8 h, 10 h or overnight.

For preparing a *nanI* complementing strain of BMC207, we first constructed pJIR750Icomp, which consists of the CN3718 *nanI* ORF, 500 bp of upstream sequence and 300 bp of downstream sequence cloned into the *C. perfringens*/*E. coli* shuttle plasmid pJIR750. A *nanJ* complementing plasmid, named pJIR751Jcomp, was similarly prepared by PCR-amplifying a product corresponding to the *nanJ* ORF, 1000 bp of upstream sequence and 400 bp of downstream sequence, and then cloning that PCR product into the *C. perfringens*/*E. coli* shuttle plasmid pJIR751. Finally, by the same method, a *nanH* complementing plasmid named pJIR751Hcomp was prepared that contains 500 bp of upstream sequence, the *nanH* ORF and 300 bp of downstream sequence cloned into pJIR751. The three sialidase complementing plasmids were then individually transformed into BMC207 by electroporation. PCR confirmed the resultant transformants contained *nanI*, *nanJ* or *nanH* wild-type genes (Figure 5A). Using primers to internal sequences of each sialidase gene and BMC207 DNA, the *nanI*, *nanJ* or *nanH* PCR products amplified large bands indicating an intron insertion. In contrast, using DNA from the three complementing strains, the PCR products amplified were of smaller size, consistent with their containing a wild-type sialidase gene, which is preferentially amplified over the larger intron-disrupted sialidase gene also present in these complementing strains.

Western blot analyses then assessed sialidase expression by the complementing strains (Figure 5B). As expected, BMC206 produced the ∼43 kDa NanH, the ∼77 kDa NanI and the ∼130 kDa NanJ. In contrast, the BMC207 null mutant expressed no sialidase proteins. These Western blots also showed that the *nanH* complementing strain BMC2073 only produced NanH, the *nanI* complementing strain BMC2072 only made NanI, and the *nanJ* complementing strain BMC2071 only expressed NanJ (Figure 5B).

The sialidase activity of the complementing strains was also assessed (Figure 5C). When sialidase activity was measured in 2 h, 4 h, 6 h, 8 h, 10 h or overnight TH cultures, the *nanI* complementing strain BMC2072 started expressing NanI as early as 2 h in TH culture. In contrast, BMC206 and the *nanJ* complementing strain began producing sialidase at 4 h. Wild-type CN3718 also expressed sialidase starting at 4 h (data not shown). The NanI complementing strain BMC2072 produced more sialidase activity than other complementing strains. Since the BMC2072 *nanI* single complementing strain already expressed more sialidase activity than either CN3718 or BMC206, and also due to technical limitations, no double or triple sialidase complementing strains were prepared.

### Culture supernatants containing NanI enhance epsilon toxin binding to MDCK cells

The availability of sialidase mutants and complementing strains allowed us to evaluate whether specific sialidases can enhance ETX binding to MDCK cells at natural sialidase expression levels and in the background of other secreted *C. perfringens* products. The same 5 µg/ml amount of AF488-pETX was added to concentrated supernatants from BMC206, the BMC207 triple sialidase null mutant strain, or the three BMC207 sialidase complementing strains (i.e. BMC2071, BMC2072 and BMC2073). Note that, i) a natural 1× concentration of *C. perfringens* culture supernatant was finally present in these MDCK cell cultures, ii) a 5 µg/ml ETX concentration corresponds to a typical natural supernatant concentration of this toxin for ETX-producing *C. perfringens* strains [5], [6] and iii) these binding experiments were performed at 37°C to obtain the enzymatic effects of sialidases (when present), but previous studies [13] have shown that prototoxin binding to MDCK cells is equivalent at 4°C or 37°C.

**Figure 6:**
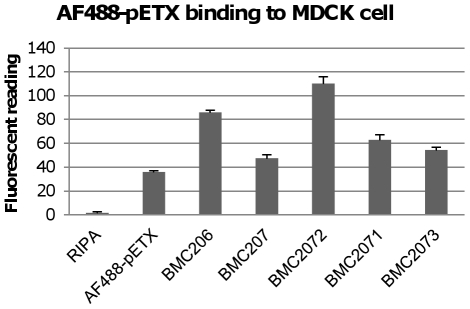
Binding of the Alex 488-labeled prototoxin to MDCK cells. The left bar shows fluorescence for a negative control sample using MDCK cells lysed with RIPA buffer but not treated with labeled prototoxin. Another control shown included fluorescence for MDCK cells treated only with labeled prototoxin binding (no *C. perfringens* culture supernatant added). BMC206, BMC207, BMC2071, BMC2072, BMC2073 samples show fluorescence measured when labeled prototoxin (5 µg/ml) was added to the indicated TH supernatant before MDCK cell treatment. The results show the average of three repetitions; the error bars indicate standard errors of the means.

When these mixtures were added to MDCK cells, the fluorescent readings showed that the BMC207 supernatant sample supported less AF488-pETX binding to MDCK cells compared against the BMC206 supernatant (Figure 6). Supernatant from the BMC2072 *nanI* complementing strain exhibited even greater levels of AF488-pETX binding than did BMC206 supernatant after a similar supplementation with the labeled prototoxin. In contrast, a smaller increase in AF488-pETX binding was noted using supernatants of the *nanJ* or *nanH* complementing strains supplemented with labeled prototoxin.

### NanI enhances epsilon toxin-mediated cytotoxicity in MDCK cells

**Figure 7:**
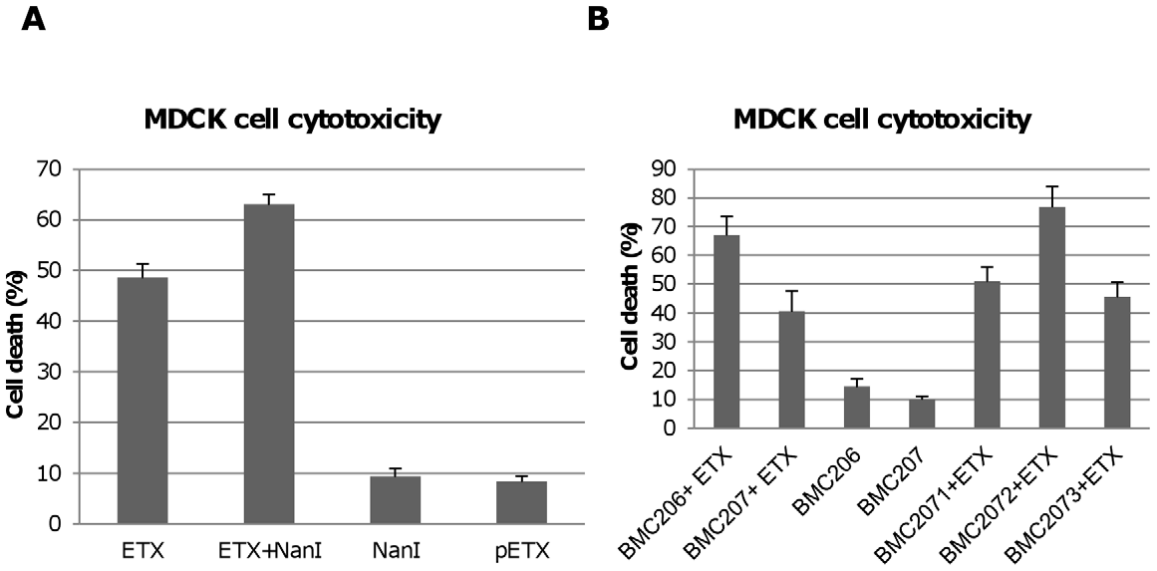
Sialidases increase cytotoxicity for MDCK cells. Panel A, percent of dead MDCK cells after treatment at 37°C for 1 h with 5 µg/ml ETX, 5 µg/ml pETX, 0.005 U/ml NanI sialidase or a mixture of 5 µg/ml ETX and 0.005 U/ml NanI sialidase, Panel B, percent of dead MDCK cells after the cells were treated for 1 h at 37°C with 5 µg/ml of ETX dissolved in BMC206, BMC207, BMC2071, BMC2072 or BMC2073 culture supernatants. BMC206 and BMC207 culture supernatants without ETX supplementation are shown for comparison. The results shown represent the average of three repetitions; the error bars indicate standard errors of the means.

Figure 1 results demonstrated that pretreating MDCK cells with purified *C. perfringens* NanI sialidase increases subsequent ETX cytotoxicity. Results shown in Figure 7A confirmed this conclusion and demonstrated that this effect is not due to direct sialidase-induced cytotoxicity.

The Figure 6 binding results suggested that, compared against supernatants from BMC207, increased MDCK cell cytotoxicity should also be observed using supernatants from BMC206 or the *nanI* complementing strain BMC2072 when those supernatants were supplemented with ETX. Verifying this suggestion, the ETX-supplemented culture supernatants of BMC206 strain caused about 30% more MDCK cell cytotoxicity than did similarly ETX-supplemented BMC207 supernatants (Figure 7B). Furthermore, complementation with the wild-type *nanI* gene substantially increased the cytotoxic effects of the sialidase-deficient BMC207 mutant, while complementation of the BMC207 mutant with the wild-type *nanJ* or *nanH* genes increased cytotoxicity to a lesser extent.

### NanI can be activated by trypsin

**Figure 8:**
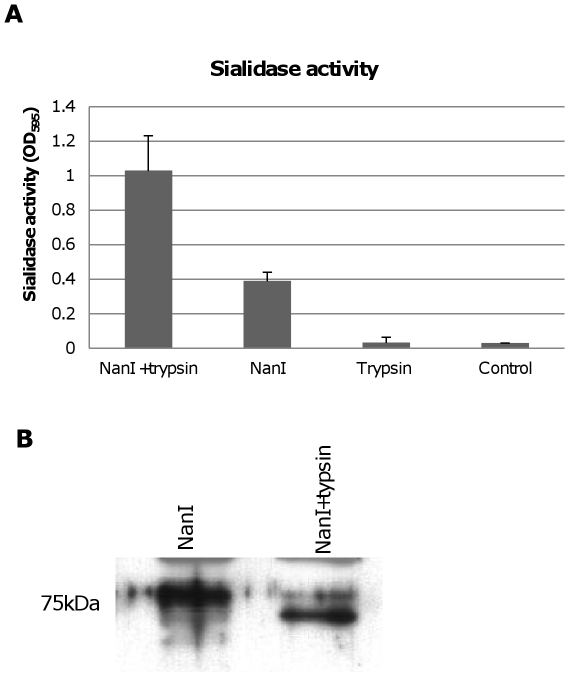
Effects of trypsin treatment on NanI sialidase. Panel A, Sialidase enzymatic activity was measured for 0.005 U/ml of purified NanI sialidase or 0.005 U/ml of purified NanI sialidase that had been treated for 1 h with 15 µg/ml of trypsin (note: following this treatment, trypsin activity was blocked using trypsin inhibitor). Also shown is sialidase activity measured for 15 µg/ml of trypsin (with trypsin inhibitor but no NanI sialidase added) and the buffer control. The results shown represent the average of three representations; the error bars indicate standard errors of the means. Panel B, Western blot comparative size analysis of NanI sialidase and trypsin-treated NanI sialidase. Size of protein in kDa is shown at left.

During natural disease, ETX is secreted into the animal intestines as an inactive prototoxin of ∼33 kDa, which can then be activated by intestinal proteases such as trypsin or chymotrypsin [11]. Interestingly, when CN3718 wild-type supernatants were trypsin-treated to activate prototoxin (data not shown), overall sialidase activity in these supernatants also significantly increased. This result was confirmed by treating purified *C. perfringens* NanI with trypsin. As shown in Figure 8A, the trypsin-treated NanI possessed significantly increased sialidase activity compared against the same amount of non-trypsin-treated NanI. When these samples were subjected to Western blotting, the results indicated that most of the trypsin-treated NanI ran as a slightly smaller protein than native NanI, supporting proteolytic processing (Figure 8B). In addition, chymotrypsin also activated NanI sialidase activity (data not shown).

### Trypsin activation of NanI can enhance cytotoxicity

**Figure 9:**
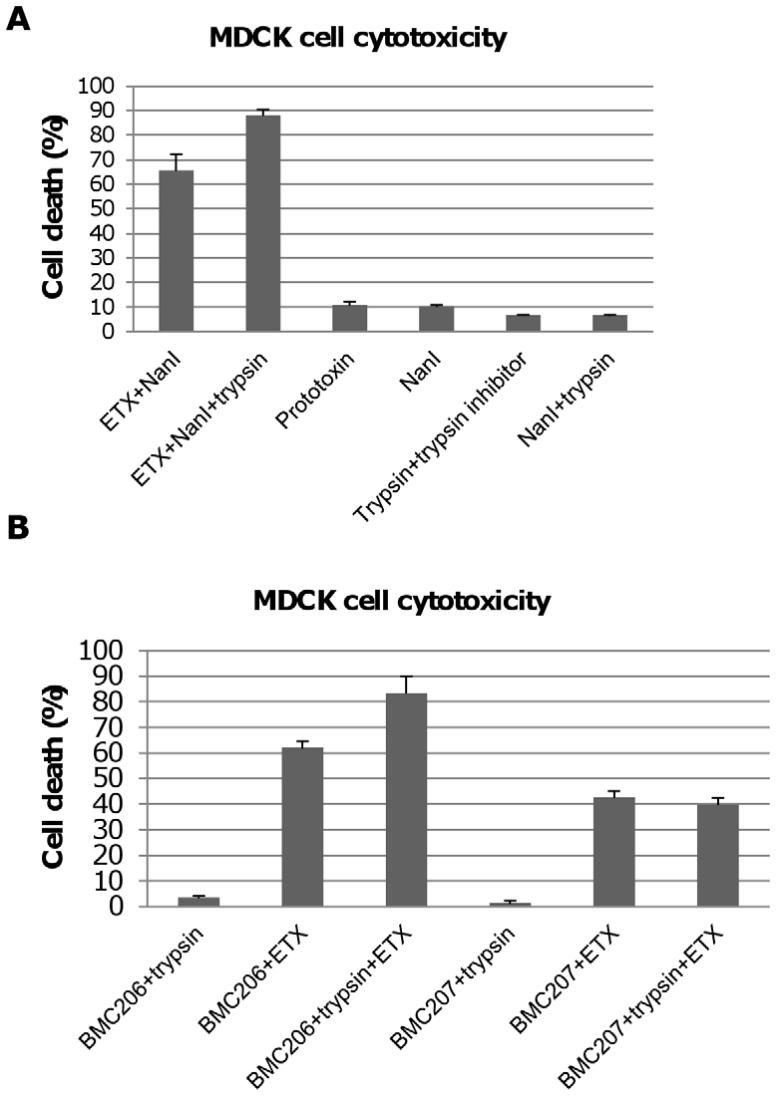
Trypsin-activation of NanI sialidase further enhances ETX-induced MDCK cell cytotoxicity. Panel A, percent of dead MDCK cells after treatment for 1 h at 37°C with 5 µg/ml of ETX in the presence of 0.005 U/ml sialidase or 5 µg/ml ETX in the presence of trypsin-treated 0.005 U NanI sialidase (note that trypsin activity in this sample was blocked with trypsin inhibitor before it was added to MDCK cells). Controls shown include: Prototoxin, NanI (no ETX), the trypsin and trypsin inhibitor mixture (no ETX or sialidase), and NanI with trypsin/trypsin inhibitor (no ETX). The results shown represent the average of three repetitions; the error bars indicate standard errors of the means. Panel B, percent of dead MDCK cells after treatment for with 5 µg/ml of ETX dissolved in BMC206 or BMC207 supernatants that had or had not been trypsin-treated for 1 h at 37°C (note: after this treatment, trypsin activity in this sample was blocked using trypsin inhibitor before the sample was added to MDCK cells). Effects of trypsin-treated BMC206 or BMC207 supernatants (no ETX addition) are shown as controls. The results shown represent the average of three repetitions; the error bars indicate standard errors of the means.

A further experiment then compared ETX cytotoxicity in the presence of purified NanI sialidase that had, or had not, been trypsin-activated prior to mixing with purified active ETX. After blocking trypsin activity from the sialidase sample with trypsin inhibitor, an enhancement of ETX cytotoxicity was observed using the trypsin-activated sialidase compared against the same amount of sialidase that had not been trypsin-activated (Figure 9A). Trypsin-activated sialidase alone caused no increase in cytotoxicity above background.

Similar experiments using concentrated *C. perfringens* culture supernatants showed that trypsin activation of these supernatants enhanced MDCK cell cytotoxicity by about 25% for BMC206 (Figure 9B). Further supporting the involvement of sialidases in this trypsin-induced increase in the cytotoxic properties of BMC206, similar trypsin treatment of BMC207 supernatants did not increase ETX-induced cytotoxicity (Figure 9B).

**Figure 10:**
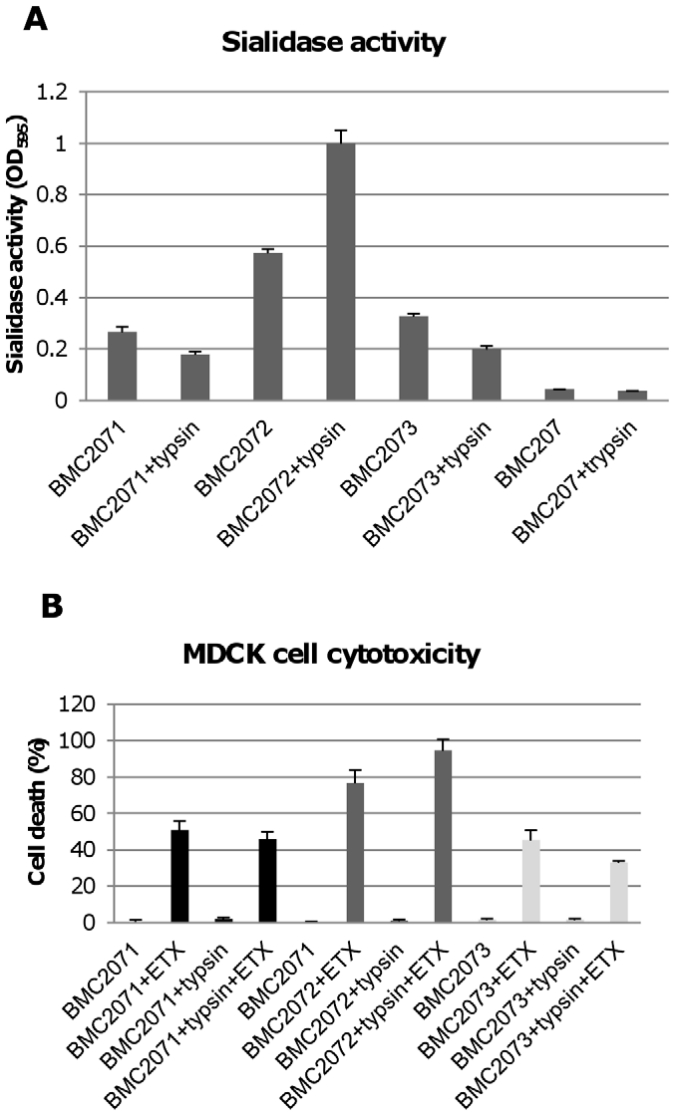
Sialidase activity and cytotoxicity for MDCK cells caused by culture supernatants from complementing strains. Panel A, sialidase activity detected in 8 h TH culture supernatants with or without trypsin treatment (note: following trypsin treatment, trypsin activity was blocked with trypsin inhibitor before the sample was assayed for sialidase activity). Effects of BMC207 and trypsin-treated BMC207 8 h TH culture supernatants are shown as controls. The results shown represent the average of three repetitions; the error bars indicate standard errors of the means. Panel B, percent of dead MDCK cells after treatment for 1 h at 37°C with 5 µg/ml of ETX added to 8 h TH culture supernatants of BMC2071, BMC2072 or BMC2073; % dead MDCK cells after treatment for 1 h with 5 µg/ml of ETX added to 8 h trypsin-treated TH culture supernatants of BMC2071, BMC2072 or BMC2073 (after this treatment, trypsin activity was blocked with trypsin inhibitor before addition of the sample to MDCK cells). Also shown are the cytotoxic effects after 1 h on MDCK cells of BMC2071, BMC2072 or BMC2073 8 h TH culture supernatants without ETX supplementation, as well as trypsin treated BMC2071, BMC2072 or BMC2073 8 h TH culture supernatants without ETX supplementation. The results shown represent the average of three repetitions; the error bars indicate standard errors of the means.

The Figure 8A results indicated that NanI can be activated by trypsin. To assess whether the other two *C. perfringens* sialidase (NanJ and NanH) can also be trypsin-activated, the supernatants of *nanI*, *nanJ* and *nanH* complementing strains were similarly treated with trypsin and their sialidase activity was measured. The results obtained indicated that only the sialidase activity of NanI is enhanced by trypsin (Figure 10A). In contrast, NanJ and NanH sialidase activity slightly decreased after similar trypsin treatment (Figure 10A). Consistent with this result, trypsin increased the MDCK cell cytotoxicity of supernatants containing only NanI, but not supernatants containing only NanJ or NanH (Figure 10B).

### Contact with certain mammalian cells upregulates CN3718 exosialidase activity

**Figure 11:**
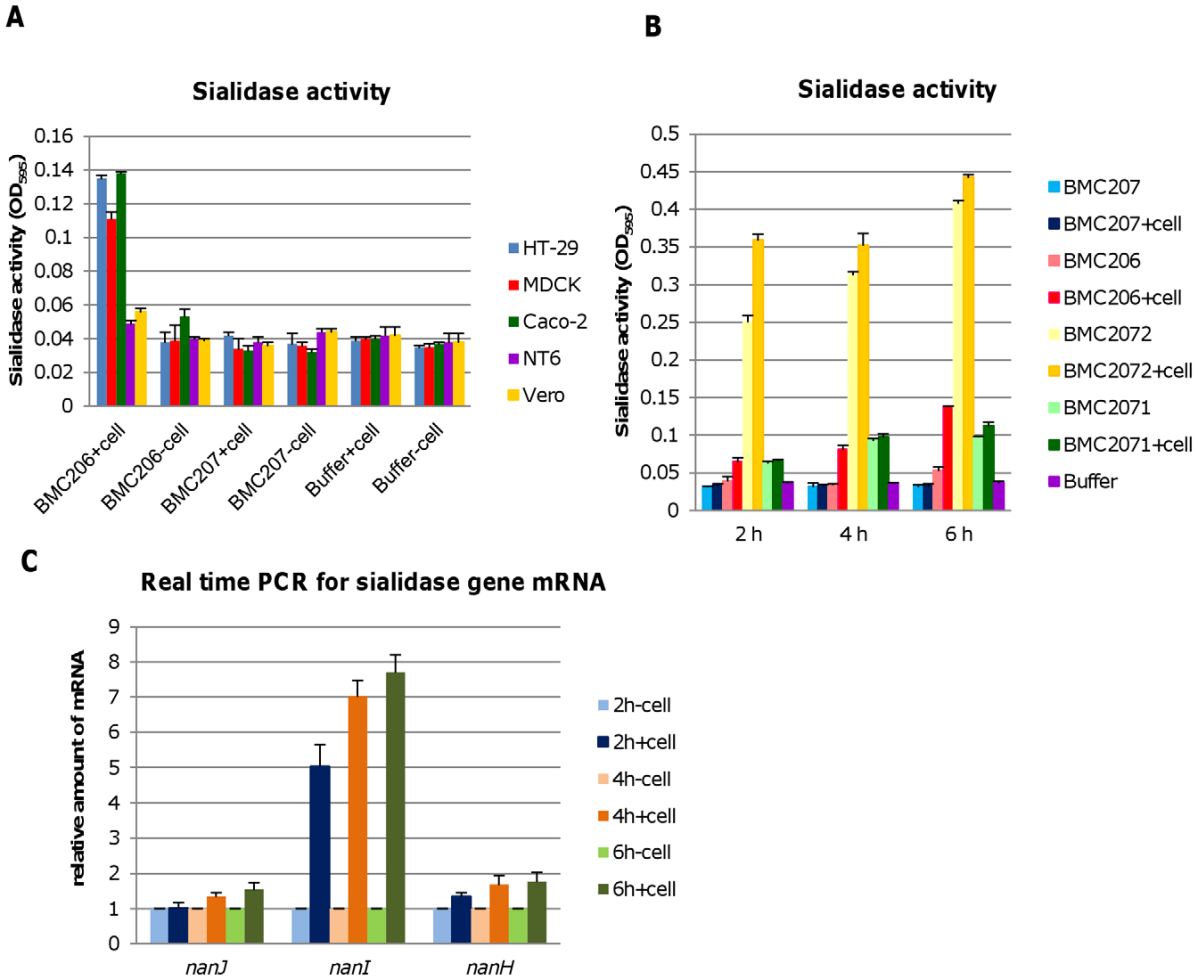
Sialidase activity upon contact with mammalian cells. Panel A, Sialidase activity detected after washed BMC206 and BMC207 cells were suspended in HBSS and added to tissue culture wells that did or did not contain monolayers of HT-29, MDCK, Caco-2, NT6 or Vero cells. Buffer w/o cells as negative control. The results show average of three representations; the error bars indicate standard errors of the means. Panel B, Sialidase activity detected after washed BMC206, BMC207, BMC2071 or BMC2072 cells were suspended in HBSS and added to tissue culture wells that did or did not contain monolayers of Caco-2 cells for 2, 4 or 6 h. The results shown represent the average of three repetitions; the error bars indicate standard errors of the means. Panel C, Real time PCR for sialidase gene mRNA expression (*nanJ, nanI* and *nanH*), when the washed bacteria were added to tissue culture wells that did or did not contain Caco-2 cells, followed by incubation for 2, 4 or 6 h. The results show the average of three representations; the error bars indicate standard errors of the means.

During natural enterotoxemias, type D strains remain present in the intestines, where they contact host enterocytes [8]. Therefore, an experiment examined whether contact with cultured mammalian cells for 6 hr (including intestinal Caco-2 and HT-29 cells as well as MDCK, Vero and NT6 fibroblasts) might affect the exosialidase activity of BMC206. This study revealed that contact between BMC206 and Caco-2, HT-29, or MDCK cells resulted in increased culture supernatant sialidase activity, although this effect was weaker using MDCK cells. In the absence of BMC206 infection, supernatant sialidase activity for Caco-2, HT-29 and MDCK cell cultures did not increase above background (Figure 11A), suggesting that the increased culture supernatant sialidase activity measured in the BMC206-infected cultures was not from secreted mammalian sialidase activity. The results also detected no culture supernatant sialidase activity upon infection of these mammalian cells with the triple sialidase null mutant BMC207. In contrast to the stimulation of sialidase activity observed using Caco-2, HT-29 or MDCK cells, exosialidase activity did not increase after BMC206 infection of Vero cell or NT6 fibroblast cultures (Figure 11A).

The nature of this host cell-induced increase in sialidase activity was further investigated using a Caco-2 cell infection model (Figure 11B). This study detected no culture supernatant sialidase activity upon infection of the triple null mutant BMC207 with Caco-2 cells up to 6 h, consistent with the increased sialidase activity detected upon BMC206 infection of Caco-2 cells involving upregulated sialidase expression by BMC206 upon Caco-2 cell contact. Results obtained using the BMC2072 NanI complementing strain suggested this upregulation of sialidase activity upon contact of BMC206 with Caco-2 cells primarily involves increased NanI production (Figure 11B). To definitively resolve whether contact with Caco-2 cells upregulates NanI expression by BMC206, quantitative RT-PCR studies were performed (Figure 11C). Those studies clearly demonstrated a substantial increase in *nanI* transcription in the presence of Caco-2 cells.

### NanI increases *C. perfringens* attachment to some mammalian cells

When causing disease originating in the intestines, *C. perfringens* type D vegetative cells likely adhere to intestinal tissue to colonize and sustain toxin production [8]. Relatively little is known about *C. perfringens* adherence to host cells, particularly enterocytes during enteritis or enterotoxemia. However, since enterocytes are coated with a variety of sialic acid-containing glycoconjugates, sialic acid residues could conceivably modulate attachment of these bacteria to the intestines.

**Figure 12:**
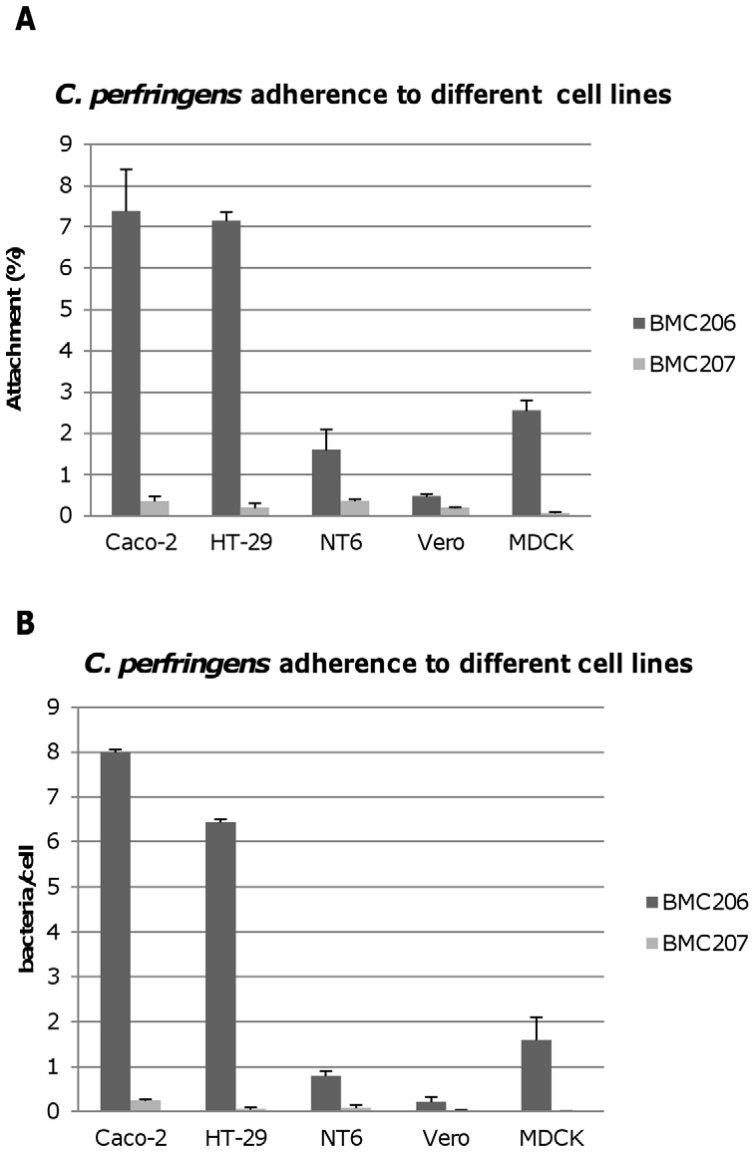
Attachment of *C. perfringens* to mammalian cells. Caco-2, HT-29, NT6, Vero or MDCK cells monolayers were incubated for 2 h with the indicated bacteria at 37°C under anaerobic conditions. Monolayers were then washed three times with HBSS buffer, lysed in distilled water and total bacteria were plated onto BHI agar plates for counting. Panel A, Attachment levels expressed as the percentage of attached bacteria relative to the total number of input bacteria. The results shown represent the average of three repetitions; the error bars indicate standard errors of the means. Panel B, Attachment levels expressed as the bacteria attached per mammalian cell. The results shown represent the average of three repetitions; the error bars indicate standard errors of the means.

Therefore, experiments tested whether BMC206 or BMC207 can attach to cultured mammalian cells, including Caco-2 cells, HT-29 cells, MDCK cells, Vero cells and NT6 fibroblasts. As shown in Figure 12A and 12B, BMC206 attached well to Caco-2 and HT-29 cells, but substantially less well to the other surveyed host cells. BMC206 attachment to Caco-2 and HT-29 cells involved sialidase production since BMC207 failed to attach to those two mammalian cell lines (Figure 12A and 12B).

**Figure 13:**
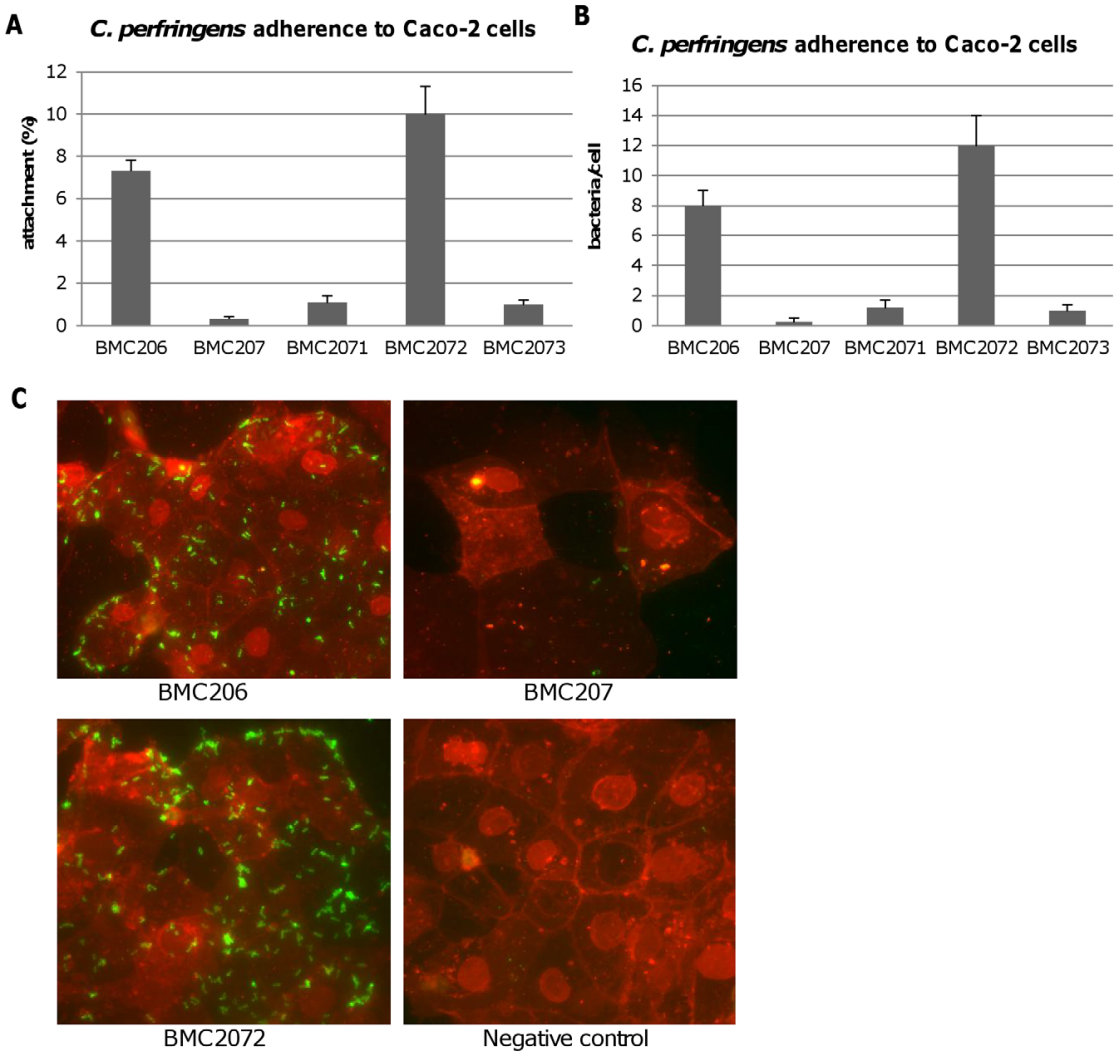
Attachment of *C. perfringens* to Caco-2 cells. Caco-2 cell monolayers were incubated for 2 h with bacteria at 37°C under anaerobic conditions. Monolayers were then washed three times with HBSS buffer, lysed in distilled water and total bacteria were plated onto BHI agar plates for counting. Panel A, Attachment levels expressed as the percentage of attached bacteria relative to the total number of input bacteria. The results shown represent the average of three repetitions; the error bars indicate standard errors of the means. Panel B, Attachment levels expressed as the bacteria attached per mammalian cell. The results shown represent the average of three repetitions; the error bars indicate standard errors of the means. Panel C, *C. perfringens* BMC206, BMC207 and BMC2072 attached to Caco-2 cells as detected by fluorescence microscopy (600×) as described in the Materials and Methods. Green: bacteria; Red: Caco-2 cells.

Additional studies were performed with the human enterocyte-like Caco-2 cell line as an *in vitro* model to examine whether sialidases play a role in CN3718 adherence to intestinal cells (Figure 13A and 13B). As already established (Figure 12A), BMC206 attached well to Caco-2 cells. In contrast, there was little or no attachment of BMC207 to these host cells under the same experimental conditions. However, the BMC2072 *nanI* complementing strain exhibited Caco-2 cell adhesion levels equal to, or exceeding those of, BMC206 (Figure 13A and 13B). In contrast, the Caco-2 adhesion ability of the *nanJ* and *nanH* complementing strains was not substantially increased over the attachment of BMC207 (Figure 13A and 13B).

A role for NanI sialidase in modifying the Caco-2 cell surface to increase the adherence of CN3718 vegetative cells to these host cells was further supported by results of an experiment where Caco-2 cells were pretreated with purified NanI. This pretreatment substantially increased the Caco-2 cell adherence of the BMC207 mutant (data not shown).

Phase-contrast and immunofluorescence microscopy analyses (Figure 13C) confirmed the strong adhesion of BMC206 or BMC2072 vegetative cells to Caco-2 cells. This microscopy detected many adherent bacteria attached to Caco-2 cells, often along their edges. In contrast, few (if any) BMC207 cells were adherent to Caco-2 cells.

## Discussion

A recent study [20] showed that a *nanI* mutation nearly completely abolished the sialidase activity of *C. perfringens* type A strain 13, which produces NanI and NanJ. However, unlike strain 13, many *C. perfringens* strains can also produce NanH [14]. Using a combination of *nanI*, *nanJ* or *nanH* single null mutants, a *nanI*/*nanJ* double null mutant, and a sialidases triple null mutant, the current study evaluated the relative contribution of each sialidase to the exosialidase activity and total sialidase activity of *C. perfringens* type D strain CN3718. These analyses revealed that, using the sialidase assay conditions employed in this study, i) CN3718 produces all three recognized *C. perfringens* sialidases but no additional unknown sialidases, ii) NanI is responsible for most exosialidase activity of this strain and iii) NanH is also a major contributor to the total sialidase activity possessed by CN3718. Furthermore, by demonstrating that CN3718 produces all three sialidases, the current results argue against proposals [17] that only myonecrosis strains of *C. perfringens* produce all three sialidases. Bioinformatics analyses of database sequences support carriage of three sialidase genes as the norm for *C. perfringens* strains, regardless of origin. In addition to our current CN3718 findings, analysis of the 8 completed or partially-completed *C. perfringens* genomes revealed that all three sialidase genes are present in 6 of those 8 isolates, which include several isolates from humans or livestock suffering from *C. perfringens* disease originating in the intestines. The two exceptions are strain 13 and SM101 that, respectively, lack *nanH* or both *nanI* and *nanJ* [14], [16].

Previous studies with other bacterial pathogens showed that sialidases can contribute to virulence in several ways, e.g., by exposing sites to facilitate more bacterial adhesion or toxin binding to host cells [19], [26]. Our results indicated that NanI sialidase increases the ETX sensitivity of MDCK cells. The mechanism of this enhancement was shown, for the first time, to involve an increase in toxin binding, suggesting NanI exposes additional ETX receptors on the host cell surface. Alternatively, NanI could modify host cell surface nonreceptors so they acquire ETX binding ability, similar to the situation in *Vibrio cholerae*, where a sialidase modifies glycolipids to increase cholera toxin binding [18]. We also demonstrated that increased ETX binding to NanI-exposed MDCK cells leads to more ETX complex formation, which translates to greater pore formation and host cell death [23]. Overall, these conclusions are consistent with, and explain for the first time, observations from a previous study reporting that *C. perfringens* sialidases can increase ETX cytotoxicity towards MDCK cells [21]. They are also consistent with previous results showing that soluble sialic acid cannot inhibit ETX binding to, or action on, MDCK cells [21]. However, the current results apparently contrast with another study [22] reporting that sialidases lower ETX binding to brain synaptosomal membrane vesicles. It is not immediately clear whether the varying conclusions amongst these studies are attributable to using MDCK cells vs. synaptosomal membranes or involve some other explanation.

Sialidases assist the ability of some pathogens to adhere to host cells or tissues. For example, sialidase contributions to adherence and colonization are well-documented for *S. pneumoniae* [19]. However, prior to the current work, only one study had addressed *C. perfringens* vegetative cell adherence to the mammalian intestinal epithelium, despite the likely importance of this process for pathogenesis; that earlier study suggested a putative *C. perfringens* collagen adhesion protein (CNA) might contribute to porcine enteritis by promoting adhesion of this bacterium to damaged intestinal tissue [27]. Our study now reports the first evidence that adherence of a *C. perfringens* strain to certain host cells, including enterocyte-like Caco-2 cells, is facilitated by sialidases, especially NanI. Inactivation of NanI sialidase production decreased adhesion of CN3718 strain by at least 20-fold and this effect was reversible by complementation. Furthermore, pretreating Caco-2 cells with purified NanI allowed adherence of the BMC207 mutant unable to produce any sialidase, indicating that NanI modifies the Caco-2 cell surface to render it favorable for CN3718 adherence. NanH and NanJ appeared to play a lesser role in facilitating *C. perfringens* adherence to Caco-2 cells based upon complementation results using the triple sialidase mutant. The varying ability of the three sialidases to enhance ETX binding or *C. perfringens* adherence likely reflects the reported enzymatic difference in sialidase kinetic properties and substrate specificities [17], although further comparative study of the properties of *C. perfringens* sialidases is warranted.

These *in vitro* findings open the possibility that sialidases, particularly NanI, contribute to virulence by facilitating *C. perfringens* adherence in the intestines, a hypothesis that should be tested in animals. In addition, future studies should identify the adhesins mediating CN3718 binding to certain host cells. Beyond the CNA study [27] mentioned in the preceding paragraph, the only other available information regarding molecular mechanisms of *C. perfringens* adhesion to host cells is a report implicating type IV pilin in type A strain 13 attachment to myoblasts [28]. Future studies will examine whether the type IV pilus or CNA protein contribute to CN3718 adherence to host cells.

A notable finding of the current work was that CN3718 adhesion is specific for certain mammalian cells. Notably, this strain adheres well to Caco-2 and HT-29 cells, both of which are intestinal cell lines. Much lower attachment of this strain was detected using cell lines of nonintestinal origin, including MDCK cells (canine kidney cells), Vero cells (African green monkey kidney cells) or NT6 rat fibroblasts cells. Furthermore, contact of CN3718 with the intestinal cells lines, and MDCK cells to a lesser degree, also stimulated *nanI* transcription. Collectively, these results suggest that CN3718, a type D disease strain, is well-adapted for sensing the presence of enterocytes and then responding by producing more sialidase to facilitate its specific attachment to these cells. However, a survey including many additional cell lines and *C. perfringens* strains are needed to test this hypothesis.

The current study also suggests that trypsin could be a greater contributor to type D disease than previously appreciated [29]. It is well established that, following secretion, the inactive ETX prototoxin must be activated by protease-induced removal of residues from the N- and C-terminus [29]. This prototoxin activation normally occurs in the gut of infected animals, where it likely involves intestinal proteases, including trypsin. Interestingly, the current study found that trypsin proteolytically activates NanI as well as ETX, thereby increasing sialidase activity. Since we also showed that trypsin activation of NanI enhances ETX binding and cytotoxicity towards MDCK cells, trypsin-activated NanI could similarly increase ETX action *in vivo*. Furthermore, since NanI was also determined to be important for CN3718 adherence to Caco-2 cells, trypsin activation of NanI could also contribute to type D disease by promoting *C. perfringens* colonization in the intestines. Chymotrypsin also activated NanI sialidase activity (data not shown), so that intestinal protease could further enhance disease.

Another possible virulence role for sialidases is suggested by our observation that various CN3718 sialidase mutants, particularly *nanI* mutants, exhibited significantly decreased ETX production levels despite wild-type growth properties (data not shown). This result might indicate that sialic acid signals ETX production, but initial attempts to prove this hypothesis have yielded inconclusive results (data not shown). Interestingly, previous studies showed that inactivating the *nanI* gene in strain 13 also affects toxin production levels [20]. However, for that strain, the *nanI* mutant showed increased production levels of alpha-toxin and perfringolysin O. Whether the increased production of alpha toxin and perfringolysin O by strain 13 involves a sialic acid signal should also be evaluated in the future.

While our findings suggest NanI plays, at minimum, two virulence roles for CN3718, i.e., enhancing both ETX binding and attachment of this strain to enterocytes, it remains unclear why this strain also produces NanJ and NanH sialidases. As mentioned, NanH and NanI have different temperature optimums, kinetic properties and substrate specificities [17]; the enzymatic characteristics of NanJ, which was only discovered during genome sequencing studies [14] remains uncharacterized. Possibly NanH and NanJ are important for growth or survival in specific environments such as soil or sewage or in other infections. Furthermore, NanJ possesses additional domains of unknown function that are missing from NanI [17]; those additional NanJ domains might contribute to growth or virulence under conditions that remain to be identified.

In summary, our *in vitro* results suggest that, while NanI sialidase does not appear to be involved in the pathogenesis of type A isolates during myonecrosis [20], it could contribute to type D enterotoxaemia and enteritis in at least two ways. First, NanI (particularly after trypsin activation in the intestines) could increase ETX binding and complex formation, thereby potentiating ETX cytotoxicity. The potential relevance of this effect for damage to non-intestinal target organs, such as brain and kidneys, is unclear since it has not yet been evaluated whether NanI sialidase can be absorbed into the circulation during type D enterotoxemias. Similarly, ETX causes limited damage in the intestines of sheep [8], so sialidase potentiation of ETX may have less importance during ovine type D disease. However, sialidases could possibly increase ETX binding to the intestines of sheep or goats and thus facilitate absorption of this toxin into the circulation. Moreover, ETX does substantially damage the caprine gastrointestinal tract [8], [30], so, by enhancing ETX intestinal toxicity, trypsin-activated NanI could directly contribute to caprine type D enteritis. A second possible virulence contribution of NanI may be to increase *C. perfringens* vegetative cell adherence to the intestinal epithelium, thereby contributing to type D infections by facilitating colonization of ETX-producing bacteria in the intestines. Lastly, our results suggest that contact with intestinal cells may stimulate *C. perfringens* to produce more NanI, thereby further potentiating (particularly after trypsin and chymotrypsin activation) ETX effects and bacterial adherence during disease. The relevance of these *in vitro* findings for possible sialidase virulence contributions should now be tested experimentally in animal models.

## Materials and Methods

### Bacterial strains and medium

CN3718, an ETX-positive *C. perfringens* type D animal disease strain (Table 1), originated from the Burroughs-Wellcome collection and was obtained via Dr. R. G. Wilkinson [5]. NCTC8346, another type D animal disease isolate, was used for ETX toxin purification [5]. *E.coli* Top10 cells (Invitrogen) were used as the cloning host. Mutant, complementing strains, and plasmids used in this study are listed in Table 1.

**Table 1:** Strains, mutants and plasmids used in this study.

Strain or plasmid	Description	Origin
**Isolate name**		
CN3718	Wild type (*etx* ^+^, *nanJ* ^+^, *nanI* ^+^, *nanH* ^+^)	Animal disease
BMC201	CN3718::*nanJ*	This study
BMC202	CN3718::*nanI*	This study
BMC203	CN3718::*nanH*	This study
BMC204	CN3718::*nanJ/nanI*	This study
BMC205	CN3718::*nanJ/nanI/nanH*	This study
BMC206	CN3718::*etx*	This study
BMC207	CN3718::*nanJ/nanI/nanH/etx*	This study
BMC2071	BMC207+ pJIR751*nanJ*	This study
BMC2072	BMC207+ pJIR750*nanI*	This study
BMC2073	BMC207+ pJIR751*nanH*	This study
**Plasmid name**		
pJIR750	*E.coli*-*C. perfringens* shuttle vector	Bannum and Rood [37]
pJIR751	*E.coli*-*C. perfringens* shuttle vector	Bannum and Rood [37]
pJIR750nanJi	pJIR750 with *nanJ* targeted intron	This study
pJIR750nanIi	pJIR750 with *nanI* targeted intron	This study
pJIR750nanHi	pJIR750 with *nanH* targeted intron	This study
pJIR750etxi	pJIR750 with *etx* targeted intron	This study
pJIR751Jcomp	pJIR751 with *nanJ* complementation fragment	This study
pJIR750Icomp	pJIR750 with *nanI* complementation fragment	This study
pJIR751Hcomp	pJIR751 with *nanH* complementation fragment	This study

Media used in this study for culturing *C. perfringens* included FTG medium (fluid thioglycolate medium; Difco Laboratories); TH medium (Bacto Todd Hewitt Broth [Becton-Dickinson], with 0.1% sodium thioglycolate [Sigma Aldrich]); TGY medium (3% tryptic soy broth [Becton-Dickinson], 2% glucose [Fisher scientific], 1% yeast extract [Becton-Dickinson], and 0.1% sodium thioglycolate [Sigma Aldrich]) and BHI agar plates (brain heart infusion, Becton-Dickinson). For culturing *E. coli*, Luria-Bertani (LB) broth (1% tryptone [Becton-Dickinson], 0.5% yeast extract [Becton-Dickinson], 1% NaCl [Fisher scientific] and LB agar (1.5% agar [Becton-Dickinson]) were used. All antibiotics used in this study were purchased from the Sigma-Aldrich Chemical Company or Fisher Scientific Company.

### Proteins and antibodies

Purified *C. perfringens* neuraminidase (NanI) was purchased from Roche Applied Science. ETX prototoxin was purified to homogeneity using a previously described method [5]. Following the method described by the manufacturer, the purified ETX prototoxin was fluorescently-labeled using an Alexa Fluor 488 protein labeling kit (Invitrogen), creating AF488-pETX. For ETX detection by Western blots, an ETX-specific monoclonal antibody (5B7; kindly provided by Paul Hauer, Center for Veterinary Biologics, Ames, Iowa) was used as primary antibody, followed by rabbit anti-mouse immunoglobulin G (IgG)-peroxidase conjugate (Sigma) as a secondary antibody. A polyclonal rabbit antibody against *C. perfringens* neuraminidases was purchased from Thermo Scientific.

### Construction of *nanJ*-, *nanI*- and *nanH*-null mutant of *C. perfringens* CN3718

The *nanJ*, *nanI* and *nanH* genes of CN3718 were each inactivated by insertion of a group II intron via the *Clostridium*-modified TargeTron gene knock-out system [24]. Using intron insertion sites identified by the Sigma TargeTron algorithm, an ∼900 bp intron was targeted into the *nanJ*, *nanI* and *nanH* ORFs in a sense orientation. The intron insertion was targeted between nucleotides 657 and 658 of the *nanJ* ORF. The primers used for PCR targeting the *nanJ* intron were 657/658-IBS, 657/658-EBS1d and 657/658-EBS2 (Table 2). The intron insertion was targeted between nucleotides 730 and 731 of the *nanI* ORF using primers 730/731-IBS, 730/731-EBS1d and 730/731-EBS2 (Table 2). The intron insertion was targeted between nucleotides 707 and 708 of the *nanH* ORF. The primers used for PCR targeting the intron into *nanH* were 707/708-IBS, 707/708-EBS1d and 707/708-EBS2 (Table 2).

**Table 2:** Primers used in this study.

Primer name	Sequence (5’→3’)	Purpose
**657/658-IBS**	AAAAAAGCTTATAATTATCCTTAACTATCGTAACTGTGCGCCCAGATAGGGTG	*nanJ* intron preparation
**657/658-EBS1d**	CAGATTGTACAAATGTGGTGATAACAGATAAGTCGTAACTAGTAACTTACCTTTCTTTGT	*nanJ* intron preparation
**657/658-EBS2**	TGAACGCAAGTTTCTAATTTCGATTATAGTTCGATAGAGGAAAGTGTCT	*nanJ* intron preparation
**730/731-IBS**	AAAAAAGCTTATAATTATCCTTAGTAAACACAGAGGTGCGCCCAGATAGGGTG	*nanI* intron preparation
**730/731-EBS1d**	CAGATTGTACAAATGTGGTGATAACAGATAAGTCACAGAGCCTAACTTACCTTTCTTTGT	*nanI* intron preparation
**730/731-EBS2**	TGAACGCAAGTTTCTAATTTCGATTTTTACTCGATAGAGGAAAGTGTCT	*nanI* intron preparation
**707/708-IBS**	AAAAAAGCTTATAATTATCCTTAAAACACAGTTCCGTGCGCCCAGATAGGGTG	*nanH* intron preparation
**707/708-EBS1d**	CAGATTGTACAAATGTGGTGATAACAGATAAGTCAGTTCCTATAACTTACCTTTCTTTGT	*nanH* intron preparation
**707/708-EBS2**	TGAACGCAAGTTTCTAATTTCGATTTGTTTTCGATAGAGGAAAGTGTCT	*nanH* intron preparation
**nanJKOF**	CTGCAATTCAAGGTGTTGGTG	*nanJ* null mutant scanning and qRT-PCR
**nanJKOR**	CTTGTCTTCTAAGCTCATATCC	*nanJ* null mutant scanning and qRT-PCR
**nanIKOF**	CAAGAGTTGGTTTTGAGC	*nanI* null mutant scanning and qRT-PCR
**nanIKOR**	AAATAAGGCTGGTATTCTG	*nanI* null mutant scanning and qRT-PCR
**nanHKOF**	AATTGGATGGCTAGGTGGAGTT	*nanH* null mutant scanning and qRT-PCR
**nanHKOR**	CAGGTGCTTCCTAAATCGTGAG	*nanH* null mutant scanning and qRT-PCR
**330/331-IBS**	AAAAAAGCTTATAATTATCCTTACCATCCATGAATGTGCGCCCAGATAGGGTG	*etx* intron preparation
**330/331-EBS1d**	CAGATTGTACAAATGTGGTGATAACAGATAAGTCATGAATTATAACTTACCTTTCTTTGT	*etx* intron preparation
**330/331-EBS2**	TGAACGCAAGTTTCTAATTTCGGTTGATGGTCGATAGAGGAAAGTGTCT	*etx* intron preparation
**etxkoF**	GGTTACTATAAATCCACAAGGA	*etx* null mutant scanning
**etxkoR**	GTTAAGAGAGCTTTTCCAACAT	*etx* null mutant scanning
**nanJcomF**	CGGCggatccCAATAGACTTAGATGTTTTAGACCC (small letters show a BamHI site)	*nanJ* complementation
**nanJcomR**	GCAGgtcgacCTATCTCCTGGTCTAACATTTT (small letters show a SalI site)	*nanJ* complementation
**nanIcomF**	TAGAgtcgacACACCTTTAAGTTTTAAGAAA (small letters show a SalI site)	*nanI* complementation
**nanIcomR**	TAGCctgcagTTTTTATTACTACCCTCTGAAG (small letters show a PstI site)	*nanI* complementation
**nanHcomF**	CGGCggatccTTTATAATAATTCTAAGTCTCACC (small letters show a BamHI site)	*nanH* complementation
**nanHcomR**	GCAGgtcgacGAAAAGAGTCTGTCATTAGCAG (small letters show a SalI site)	*nanH* complementation
**16sF**	CGCATAACGTTGAAAGATGG	qRT-PCR 16S rRNA house keeping gene
**16sR**	CCTTGGTAGGCCGTTACCC	qRT-PCR 16S rRNA house keeping gene

The 350 bp intron PCR products were inserted into pJIR750ai between the HindIII and BsrGI enzyme sites in order to construct *nanJ*, *nanI* and *nanH*-specific TargeTron mutagenesis plasmids. The resultant plasmids, named pJIR750nanJi, pJIR750nanIi and pJIR750nanHi respectively (Table 1), were individually electroporated into wild-type CN3718. This produced *nanJ*, *nanI* and *nanH* single null mutants named, respectively, BMC201, BMC202 and BMC203 (Table 1).

The CN3718 transformation efficiency was ∼240 transformants/µg plasmid DNA. Transformants were selected on BHI agar plates containing 15 µg/ml of chloramphenicol. Transformant colonies were then identified by colony PCR using primers nanJKOF and nanJKOR (Table 2) for screening *nanJ*-null mutants (BMC201), primers nanIKOF and nanIKOR (Table 2) for screening *nanI*-null mutants (BMC202), and primers nanHKOF and nanHKOR (Table 2) for screening *nanH*-null mutants (BMC203). Each reaction mixture was subjected to the following PCR amplification conditions: cycle 1, 95°C for 5 min; cycles 2 through 35, 95°C for 30 s, 55°C for 40 s, and 68°C for 90 s; and a final extension for 5 min at 68°C. An aliquot (20 µl) of each PCR sample was electrophoresed on a 1.5% agarose gel and then visualized by staining with ethidium bromide. The mutants were cured of the intron-carrying donor plasmid as described [25].

### Construction of the CN3817 *nanI*/*nanJ* double null mutant BMC204

To prepare a *nanI*/*nanJ* double null mutant, pJIR750nanJi was electroporated into the BMC202 *nanI* null mutant strain, which had been cured of the pJIR750nanIi plasmid. Transformants were grown on BHI agar plates containing 15 µg/ml chloramphenicol and putative *nanJ/nanI* double null mutants were then identified by demonstrating the presence of introns in both the *nanI* and *nanJ* ORFs by PCR. The confirmed *nanI*/*nanJ* double null mutant (named BMC204, Table 1) was cured the intron-carrying donor plasmid pJIR750nanJi. The identity of this BMC204 *nanI*/*nanJ* double null mutant was then confirmed by Southern blotting, which demonstrated the presence of two introns in the mutant.

### Construction of the BMC205 *nanI*/*nanJ*/*nanH* triple null mutant

To prepare a *nanI/nanJ/nanH* triple null mutant, pJIR750nanHi was electroporated into the BMC205 double *nanI*/*nanJ* null mutant strain and transformants were then grown on BHI agar plates containing 15 µg/ml of chloramphenicol. Putative *nanH* null mutants were screened by PCR, as described above. The presence of introns in the *nanI*, *nanJ* and *nanH* genes of this mutant (named BMC205, Table 1) was confirmed by PCR and Southern blotting, following curing of the intron-carrying donor plasmid pJIR750nanHi.

### Inactivation of the *etx* gene in CN3817 and BMC205

An intron insertion, with a sense orientation, was targeted between nucleotides 330 and 331 of the *etx* ORF. The primers used for targeting this intron were 330/331-IBS, 330/331-EBS1d and 330/331-EBS2 (Table 2). The 350-bp PCR products were inserted into pJIR750ai between the HindIII and BsrGI enzyme sites in order to construct an *etx*-specific TargeTron plasmid. The resultant plasmid, named pJIR750etxi (Table 1), was electroporated into either wild-type CN3718 or BMC205 to inactivate their *etx* genes, which produced (respectively) *etx* null mutant strains named BMC206 and BMC207 (Table 1). The primers used for *etx* null mutant scanning were etxkoF and etxkoR (Table 2).

### Southern hybridization analyses

DNA was isolated from wild-type CN3718, the five sialidase null mutants (including the three single null mutants BMC201, BMC202 and BMC203, the double *nanJ/nanI* null mutant strain BMC204, and the triple sialidase null mutant strain BMC205) and the two *etx* null mutants of CN3718 and BMC205 (including the BMC206 null mutant that produces all three sialidases and the BMC207 null mutant unable to produce any sialidase) using the MasterPure^TM^ Gram-Positive DNA Purification Kit (Epicenter). Each DNA was then digested with BsrGI or EcoRI overnight at 37°C and run on a 1% agarose gel. After the alkali transfer to a nylon membrane (Roche), the blot was hybridized with a digoxigenin-labeled, intron-specific probe as described previously [25]. This intron-specific probe was prepared using the primers KO-IBS and KO-EBS1d [25] and a PCR DIG Labeling Kit (Roche Applied Science) according to the manufacturer’s instructions.

### Complementation of the *nanJ*, *nanI* and *nanH* null mutants

DNA was isolated from CN3718 using the Master Pure^TM^ Gram-positive DNA purification kit. The primers nanJcomF and nanJcomR (Table 2) were used for *nanJ* complementing strain construction. The primers nanIcomF and nanIcomR (Table 2) were used for *nanI* complementing strain construction. The primers nanHcomF and nanHcomR (Table 2) were used for construction of a *nanH* complementing strain. The PCR reactions were set up as: 1 µl of each pair of primers (at a 0.5 µM final concentration), 1 µl of purified DNA template and 25 µl 2×Taq Long Range Mixture (NEB) were mixed together and ddH_2_O was added to reach a total volume of 50 µl. The reaction mixtures were then placed in a thermal cycler (Techne) and subjected to the following amplification conditions: 1 cycle of 95°C for 2 min, 35 cycles of 95°C for 30s, 55°C for 40s, and 65°C for 5 min, and a single extension of 65°C for 5 min. The resultant 4.9kb *nanJ* PCR product, 2.8 kb *nanI* PCR product, or 2.3 kb *nanH* PCR product were each separately cloned into the Invitrogen pCR2.1 TOPO vector according to the manufacturer’s instruction and inserts were then sequenced at the University of Pittsburgh Core Sequencing Facility. Using BamHI and SalI, the *nanJ* insert was removed from the TOPO vector and ligated into the pJIR751 *C. perfringens/E. coli* shuttle plasmid, forming a plasmid named pJIR751nanJcomp (Table 1). Using the same method, *nanI* and *nanH* inserts were separately cloned into pJIR750 and pJIR751 *C. perfringens/E. coli* shuttle plasmids, forming plasmids named pJIR750nanIcomp and pJIR751nanHcomp, respectively (Table 1). These plasmids were individually introduced, by standard electroporation techniques, into the BMC207 null mutant strain to create *nanJ, nanI* and *nanH* complementation strains named, respectively, BMC2071, BMC2072 and BMC2073 (Table 1).

### Western blot analyses

A 0.2 ml aliquot of an overnight FTG culture of the wild-type, null mutants or complementing strains was inoculated into 10 ml of TH medium. To perform ETX and sialidase Western blots, supernatants of 8 h or overnight TH cultures were used. Samples were collected and each supernatant (or the sonicated whole culture) was mixed with SDS loading buffer and boiled for 5 min. Those mixtures were electrophoresed on a 12% polyacrylamide gel containing SDS for analyzing ETX or an 8% polyacrylamide gel containing SDS for analyzing sialidase proteins. The gels were then subjected to Western blotting using appropriate antibodies, as previously described [31].

### Sialidase enzyme activity assay

To assay sialidase enzyme activity, a previously described protocol was used [20] Briefly, 20 µl of TH culture supernatant was added to 60 µl of 100 mM sodium acetate buffer (pH 7.2) in a microtiter tray. A 20 µl of aliquot 4 mM 5-bromo-4-chloro-3-indolyl-α-D-N-acetylneuraminic acid (Sigma) was then added, and the tray was incubated at 37°C for 30 min. The absorbance at 595 nm was then determined using a microplate reader (Bio-Rad).

### Growth rate measurement for wild type, mutant and complementing strains

A 0.1 ml aliquot of an FTG overnight culture of the wild-type strain, null mutants or complementing strains was transferred to TH medium, which was grown at 37°C overnight. A 0.2 ml aliquot of each TH overnight culture was then inoculated into 10 ml of pre-warmed TH medium and the OD_600_ of those cultures was measured at 37°C over time to determine vegetative growth, as described previously [32].

### Cell culture

Madin-Darby Canine Kidney (MDCK) epithelial cells were cultured in a 1∶1 (v/v) mix of Dulbecco’s Modified Eagle’s Medium (DMEM, Sigma) and Nutrient Mixture F12 HAM (Sigma), supplemented with 3% fetal bovine serum (Mediatech), 100 µg/ml penicillin/streptomycin (Sigma) and 1% glutamine (Sigma). Caco-2 cells were maintained in Eagle minimal essential medium (Sigma) supplemented with 10% fetal bovine serum (Mediatech), 1% nonessential amino acids (Sigma), and 100 µg/ml of penicillin/streptomycin. Vero cells were cultured in M199 medium (Sigma) supplemented with 5% fetal bovine serum and 100 µg/ml of penicillin/streptomycin. HT-29 cells were cultured in RPM1-164 medium (Sigma) supplemented with 10% fetal bovine serum and 100 µg/ml of penicillin/streptomycin. NT6 fibroblast cells were culture in Alpha-MEM (Modified Eagle Medium) supplemented with 7.5% fetal bovine serum, 2 mM L-glutamine, 1× Non Essential Amino Acids, 1 mM sodium pyruvate, and 100 µg/ml of penicillin/streptomycin. All cells were grown at 37°C with 5% atmospheric CO_2_.

### Cytotoxicity assays

Prior to use in ETX cytotoxicity assays, purified ETX prototoxin was activated by trypsin. For this purpose, 50 µl aliquots of labeled or unlabeled prototoxin (2 mg/ml) were incubated with 12.5 µg of trypsin (Sigma)/µg prototoxin for 1 h at 37°C. After that incubation, trypsin inhibitor (Sigma) (1∶1 v/v) was added to remove trypsin activity. The same trypsin/trypsin inhibitor mix (no ETX) was used as a negative control for cytotoxicity assays. Confluent monolayer MDCK cells were incubated in the presence or absence of 5 µg/ml of activated ETX; in some experiments, that same amount of activated ETX was added to a 10 × concentrated TH supernatant culture for 1 hr at 37°C. After this incubation, cytotoxicity was measured using the LDH Cytotoxicity Detection Kit (Roche).

### Trypsin or chymotrypsin treatment of NanI and culture supernatants

An aliquot of 0.005 U/ml NanI (Roche) or 8 h TH culture supernatants of BMC206, BMC207, BMC2071, BMC2072 or BMC2073 were incubated with 12.5 µg of trypsin (Sigma) or chymotrypsin (Sigma) for 1 h at 37°C. After that incubation with trypsin, trypsin inhibitor (Sigma) (1∶1 v/v) was added to remove trypsin activity from those samples. Sialidase activities were detected by the method described before [20].

### ETX binding/complex formation in MDCK cells

Confluent monolayers of MDCK cells grown in 6 well cluster plates (Corning) were or were not pretreated with 0.005 U/ml or 0.001 U/ml of *C. perfringens* sialidase (Roche) for 30, 60, or 90 min. After this treatment, 5 µg/ml of AF488-ETX was added and the cells were then further incubated for 60 min at 37°C. Following this toxin challenge, the cells were harvested and washed twice with PBS buffer and the pellets were then resuspended in 50 µl of PBS. After treatment of those asample with 1 µl of Benzonase nuclease (Novagen) for 5 min at room temperature, 12 µl of 5×SDS loading buffer was added. These samples were then electrophoresed on a SDS-containing 8% polyacrylamide gel, in the dark. The resultant gel was imaged using a Typhoon 9400 variable mode imager (Amersham Biosciences), with fluorescence emission set to detect the Alexa Fluor 488 label using the green laser with wavelength 532 nm. For detection of the molecular weight markers, the red laser was used with a wavelength of 633 nm. Complex formation was then quantified using Imagequant version 5.2 (Molecular Dynamics).

To evaluate ETX binding, confluent monolayers of MDCK cells grown in 24 well cluster plates (Corning) were pretreated with a 0.005 U/ml or 0.001 U/ml of NanI sialidase at 37°C, and then challenged with 5 µg/ml of AF488-pETX suspended in 250 µl of buffer or culture supernatants (from BMC206, BMC207, BMC2071, BMC2072, BMC2073) that had been concentrated 10× using an Ultrafiltration centrifuge tube (Millipore). After 60 min at 37°C, the cells were harvested and washed twice in PBS. The washed pellets were then resuspended in 50 µl of RIPA buffer (Invitrogen) and added to 96-well plates, where fluorescence was quantified using a Multi-mode Microplate Reader (Synergy^TM^4, BioTek).

### Analysis of sialidase production after *C. perfringens* strains contact Caco-2 cells, HT29 cells, NT6, Vero cells and MDCK cells

A 0.1 ml aliquot of an FTG overnight culture of BMC206, BMC207, BMC2071 or BMC2072 was transferred to TH medium, which was grown at 37°C overnight. A 0.1 ml aliquot of each TH overnight culture was then inoculated into fresh 10 ml TH medium. These overnight cultures were centrifuged and the pelleted cells were washed three times with HBSS buffer and then resuspended in HBSS buffer at 10^7^/ml. Confluent monolayers of Caco-2 cells, MDCK cells, Vero cells, HT-29 cells, and NT-6 cells grown in 6 well cluster plates were washed three times with HBSS and then challenged with a 1 ml aliquot of the washed bacterial cell suspension. Following anaerobic incubation at 37°C, for 2, 4, or 6 h, the supernatants were removed, centrifuged and 60 µl supernatant aliquot were used to measure sialidase activity by the method described above, except the sample was incubated at room temperature overnight instead of 37°C for 30 min before the absorbance was read in order to increase assay sensitive. Control washed bacterial cell suspensions were treated similarly except for the absence of cells.

### RNA isolation and qRT-PCR

As described previously [25], total RNA was extracted from 1 ml of a 2, 4, or 6 h culture of BMC206 that had or had not been in contact with Caco2 cells. All RNA samples were treated with DNAaseI (Promega). The purified RNA was quantified by absorbance at 260 nm and stored in a -80°C freezer.

qRT-PCR reactions were performed on the purified RNA samples using the iScript^TM^ one-step RT-PCR kit with SYBR green (Bio-Rad). Briefly, 100 ng of each RNA sample was reverse-transcribed to cDNA at 50°C for 10 min. That cDNA was then used as template for PCR reactions with primers 16sF and 16sR (Table 2) targeting the 16sRNA as a housekeeping gene; primers nanIKOF and nanIKOR targeting the *nanI* gene, primers nanJKOF and nanJKOR targeting the *nanJ* gene, or primers nanHKOF and nanHKOR targeting the *nanH* gene (Table 2). All reactions were performed at AB Appled Biosystems Step One plus Real-Time PCR system.

### Attachment assays

A 1.5 ml aliquot of a TH overnight culture of BMC206, BMC207, BMC2071, BMC2072 or BMC2073 was centrifuged and the bacterial pellet was then washed three times with HBSS buffer. After resuspension of the washed pellet in 1.5 ml of HBSS buffer, these suspensions were serially diluted from 10^-2^ to 10^-7^ with sterile water and aliquots were spread onto BHI agar plates. Following an overnight anaerobic incubation at 37°C, the colonies arising on these plates were counted to determine the number of CFU added to the mammalian cell cultures. Monolayers of Caco-2, HT-29, Vero, MDCK or NT6 fibroblasts were then incubated under anaerobic conditions with a 100 µl aliquot of these washed bacteria for 2 h at 37°C. After this incubation, the monolayers were washed with HBSS three times and host cell-associated bacteria were retrieved by lysing the monolayers in distilled water. Aliquots of these suspensions were plated onto BHI agar plates. After overnight anaerobic incubation at 37°C, the colonies arising on the plates were used to calculate the number of adherent CFU.

### Immunofluorescence microscopy

Caco-2 cells were grown to confluency in an 8-well chamber slide (Fisher). A 1 µl aliquot of a BMC206, BMC207 and BMC2072 overnight TH culture was added to each chamber of the slide, and the slide was then incubated at 37°C for 2 h. Cells were washed three times with HBSS buffer. The slides were fixed in 4% formaldehyde in HBSS for 30 min at room temperature. The fixed cells were incubated with a 1∶500 dilution of anti-*C. perfringens* rabbit polyclonal antibody (Genway) in HBSS with 10% BSA for 2 h at room temperature. Those cells were then washed three times with HBSS buffer and incubated with 1∶500 Alexa Fluor-488 goat anti-rabbit IgG (Invitrogen) in HBSS with 10% BSA for 1 h at room temperature. After 3 more washes with HBSS buffer, the cells were incubated with 1∶200 Vybrant^TM^ Cell-labeling solutions (Molecular Probes) for 30 min at room temperature. Following a final three washes with HBSS buffer, the chambers were removed, and a coverslip was mounted with Fluoro-Gel (Electron Microscopy Sciences). Imaging was performed on a Zeiss Axioskop 40 immunofluorescence microscope.
